# A model for Crohn’s disease post-ileal resection fibrosis development using human ileal enteroids and myofibroblasts

**DOI:** 10.3389/fphys.2026.1764088

**Published:** 2026-04-15

**Authors:** Jun Hwan Yoo, Yoon Jeong Choi, Iryna V. Pinchuk, Don W. Powell, Nicholas C. Zachos, Mark Donowitz

**Affiliations:** 1 Department of Medicine, Division of Gastroenterology and Hepatology, the Johns Hopkins University School of Medicine, Baltimore, MD, United States; 2 Department of Gastroenterology, CHA Bundang Medical Center, CHA University School of Medicine, Seongnam, Republic of Korea; 3 Department of Medicine, Penn State Health Milton S. Hershey Medical Center, Hershey, PA, United States; 4 Department of Internal Medicine, Division of Gastroenterology and Hepatology, UTMB, Galveston, TX, United States; 5 Department of Physiology, the Johns Hopkins University School of Medicine, Baltimore, MD, United States

**Keywords:** conditioned medium (CM), epithelial-mesenchymal crosstalk, intestinal fibrosis, stricture formation, TNF

## Abstract

**Background:**

Fibrosis and inflammation frequently emerge soon after ileal resection for strictures in Crohn’s disease (CD).

**Objective:**

This study examined the profibrotic and proinflammatory effects of conditioned media from TNF and IFN-γ (T+I)-exposed healthy and inactive CD ileal enteroids (CDiE) on subepithelial myofibroblasts (SEMF), modeling early post-ileal resection conditions in CD.

**Methods:**

Ileal enteroids from healthy subjects (HE) and inactive CD patients (CDiE) were exposed to T+I for 6 hours, followed by an 18-hour conditioning period. Conditioned media were collected and applied to human intestinal myofibroblasts (HIMF) from healthy and inactive CD ileum for 24 hours to assess proinflammatory and profibrotic gene expression.

**Results:**

T+I exposure induced epithelial damage and cell death in CDiE, while reducing proliferation and decreasing occludin and F-actin expression in both HE and CDiE. TEER (transepithelial electrical resistance) declined in CDiE but remained stable in HE. Conditioned media from T+I-exposed enteroids altered HIMF-HC (healthy control) and HIMF-CD mRNA expression, upregulating proinflammatory genes (*IL1B*, *IL6*, *MCP1*, and *IKBA*) while downregulating profibrotic genes (*COL1A1*, *ACTA2*, *TGFB*, and *SRF*).

**Conclusion:**

Cytokine-exposed HE- and CDiE-derived conditioned media promoted inflammation while suppressing fibrosis-associated gene expression in subepithelial myofibroblasts. Our results suggest that damaged intestinal crypts in early CD inflammation may not directly trigger certain fibrogenic markers *in vitro*. This model of the early post-ileal resection state provides a platform to identify key targets for preventing fibrosis progression and potentially reducing the need for repeat ileal surgery in CD patients.

## 1 Introduction

Human intestinal subepithelial myofibroblasts (SEMF) are the pivotal cells which contribute to fibrogenesis in the intestine (Powell et al., 1999). SEMF are positioned close to both the epithelium and immune cells of the lamina propria, interacting with both, either directly or via soluble mediators (Powell et al., 1999; Bamias et al., 2017). Soluble factors secreted by SEMF, such as Noggin and R-spondin, also modulate key components of the intestinal stem cell niche, thereby influencing epithelial proliferation (Roulis and Flavell, 2016). However, the effects of soluble factors released from healthy and damaged intestinal epithelial cells (IECs) on SEMF require further evaluation.

It has been hypothesized that epithelial cells, once damaged, stimulate fibrogenesis via the activation of adjacent myofibroblasts. This process is particularly likely to occur in organs where myofibroblasts and epithelial cells normally coexist in close proximity, such as in the lung, kidney, liver, skin, and peritoneum, as well as in the intestine (Sakai and Tager, 2013). In the kidney, the damaged renal tubular epithelial cells secrete profibrotic mediators, including TGF-β1, connective tissue growth factor (CTGF), and sonic hedgehog (Shh), and these mediators subsequently activate interstitial fibroblasts, leading to the renal fibrosis (Prunotto et al., 2012; Tan et al., 2016). In addition, the increased secretion of profibrotic mediators, such as TGF-β1 and CTGF, and the decreased secretion of antifibrotic prostaglandin E_2_ (PGE_2_) from damaged alveolar epithelial cells contributes to the progression of idiopathic pulmonary fibrosis (Sakai and Tager, 2013). In the skin, direct co-culture with keratinocytes activates fibroblasts through mechanisms dependent on TGF-β1, endothelin-1 (ET-1), and IL-1α (Shephard et al., 2004; Aden et al., 2010). In intestine, necrotic epithelial cell derived IL-1α induces proinflammatory cytokine (IL-6, IL-8) production in human intestinal fibroblasts, and is a mechanism by which the intestinal inflammatory response can be amplified and maintained (Scarpa et al., 2015). Proinflammatory cytokines (IL-1α, TNF, and IFN-γ) induced TGF-β1 in colonic epithelial cell lines (HT29 and CaCO-2). Conditioned media from these damaged epithelial cell lines induced collagen production in colonic subepithelial myofibroblasts through TGF-β1, CTGF, and ET-1 independent pathways (Drygiannakis et al., 2013).

In this study, we investigated the potential proinflammatory and profibrotic impact of IECs on neighboring intestinal SEMF. To model the Th1-mediated inflammation characteristic of Crohn’s disease (CD), we induced proinflammatory damage in ileal enteroids derived from healthy controls and patients with inactive CD using TNF and IFN-γ. Enteroids derived from inactive regions of the CD ileum were utilized to model the early post-ileal resection stage – prior to the recurrence of clinical inflammation – to investigate whether epithelial damage alone could elicit fibrotic responses in HIMF in the absence of active inflammation (Rutgeerts et al., 1990). We then examined the influence of the conditioned media from these damaged enteroids by accessing the RNA expression of both proinflammatory and profibrotic genes in SEMFs derived from healthy and inactive CD ileum.

## 2 Materials and methods

### 2.1 Human specimens

De-identified human ileum was obtained from healthy subjects and from patients with active and inactive CD who provided informed consent (**Table 1**). All experimental methods were approved by the Johns Hopkins University Institutional Review Board (IRB# NA_00038329). The ileal enteroid lines studied included four enteroid lines from healthy subjects, one line from a patient with active ileal CD and one from a patient with inactive CD. Intestinal biopsies were collected from the ileum of healthy subjects who underwent screening colonoscopy for colorectal cancer or gastrointestinal symptoms and were confirmed to have a histologically normal ileum. Human ileal subepithelial myofibroblasts were isolated from full thickness ileum from a healthy control (HIMF-HC) and from a patient with inactive CD (HIMF-CD). The enteroid and HIMF lines used in this study were derived from independent, non-donor-matched individuals. All human samples and donor information were de-identified in accordance with the IRB protocols. Consequently, clinical data were limited to age, sex, and ethnicity for certain lines, and detailed medical histories (e.g., medication or surgical history) were not accessible due to privacy regulations.

**Table 1:** Clinical descriptions and origins of human ileal tissues (biopsies and surgical specimens).

Lines, name of line	Clinical description	Origin
HE, 60I	Healthy	Ileum
HE, 46I	Healthy	Ileum, 50/M, African
HE, 109I	Healthy	Ileum
HE, 104I	Healthy	Ileum
CDiE, 69I	Crohn’s disease, inactive site	Ileum, 39/F, Asian
CDaE, 68I	Crohn’s disease, active site	Ileum, 39/F, Asian
HIMF-HC	Healthy	Ileum
HIMF-CD	Crohn’s disease, inactive site	Ileum

Demographics (age, sex, and ethnicity) for certain lines were not available from the source/provider.

### 2.2 Generation of human ileal enteroids

Enteroids were established from crypts isolated from ileal biopsies or surgical specimens from healthy subjects or CD patients using the protocol of Sato et al (Sato et al., 2011; Sato and Clevers, 2013), with minor modifications, as previously described (Foulke-Abel et al., 2016; Yin et al., 2018; Singh et al., 2022). Enteroids were embedded in Matrigel (Corning, Tewksbury, MA) in expansion medium composed of advanced Dulbecco’s modified Eagle medium/F12 (Life Technologies, Carlsbad, CA) containing 100 U/mL penicillin/streptomycin (Quality Biological, Gaithersburg, MD);10 mmol/L HEPES (Life Technologies); and 1× GlutaMAX (Life Technologies); with 50% Wnt3A-conditioned medium (produced by L-Wnt3A cell line, ATCC CRL-2647); 15% R-spondin 1-conditioned medium (produced by HEK293T cell line stably expressing mouse R-spondin 1 (kindly provided by Calvin Kuo, Stanford University, Stanford, CA)); 10% Noggin-conditioned medium (produced by HEK293T cell line stably expressing mouse Noggin); 1×B27 supplement (Life Technologies); 1 mmol/L N-acetylcysteine (Sigma-Aldrich); 50 ng/mL human epidermal growth factor (Life Technologies); 1 µg/mL (Leu-15) gastrin (AnaSpec, Fremont, CA); 500 nmol/L A83-01 (Tocris, Bristol, United Kingdom); 10 µmol/L SB202190 (Sigma-Aldrich); and 100 µg/mL Primocin (InvivoGen, San Diego, CA). Enteroids were cultured in a 5% CO2 atmosphere at 37 °C and passaged every 7–12 days. Expansion medium was supplemented with 10 µmol/L Y-27632 (Tocris) and 10 µmol/L CHIR99021 (Tocris) during the first 2 days after passaging. The 3D enteroids are shown in **Supplementary Figure 1A**.

### 2.3 Preparation of enteroid monolayers

Enteroid monolayers were formed as previously described (In et al., 2016; Noel et al., 2017; In et al., 2019). In short, Transwell inserts (polyester membrane with 0.4-µm pores; Corning) were coated with 33 µg/mL (10 µg/cm^2^) human collagen IV solution (Sigma-Aldrich) and incubated at 37˚C for at least 2 hours. Organoids were harvested, triturated, and added to the top of Transwell inserts after being resuspended in expansion medium. Each Transwell insert received approximately 50–100 organoid fragments and was maintained with 100 uL expansion media in the upper chamber and 600 uL in the lower chamber. Organoid monolayers were cultured in a 5% CO_2_ atmosphere at 37˚C. Expansion media were supplemented with Y-27632 and CHIR99021 during the first 2 days after seeding. Formation of organoid monolayers was monitored by morphologic observation using a Zeiss AXIO inverted microscope (Carl Zeiss, Thornwood, NY) and by transepithelial electrical resistance (TEER) measured by the epithelial volt/ohm meter (EVOM^2^; World Precision Instruments, Sarasota, FL). The resistance of the monolayers is expressed as Ω.cm^2^ with normalization to the surface area of the Transwell inserts (0.33 cm^2^). The confluence of enteroid monolayers was determined based on the level of the TEER. The percentage change in TEER was calculated using the equation below, as previously described in (Ranganathan et al., 2019): Percentage Change in TEER = ((TEER_exposure or exposure + conditioning_) – (TEER_baseline_))/(TEER_baseline_) × 100%.

### 2.4 Human ileal myofibroblasts: isolation and culture

Primary HIMF were isolated from surgical resection specimens and cultured with some modifications as previously described (Strong et al., 1998; Choi et al., 2019, 2024). HIMF-CD were derived from the ileal segments of a patient undergoing surgical resection for intestinal strictures due to CD. HIMF-HC was isolated from macroscopically and histologically normal ileal tissues obtained from a patient undergoing right hemicolectomy for colorectal cancer. Briefly, HIMF were derived from outgrowths of minced ileal mucosal explants. These explants were cultured on etched polystyrene flasks using a growth medium comprised of Dulbecco’s modified Eagle’s medium/high glucose (Hyclone, Logan, UT); 10% fetal bovine serum (American Type Culture Collection, Manassas, VA); 4 mmol/L L-glutamine (Gibco, Carlsbad, CA); 25 mmol/L HEPES; 100 U/mL penicillin; 100 μg/mL streptomycin; and 0.25 μg/mL amphotericin B (all from Lonza, Walkersville, MD). The cells were utilized between passages 6 and 10 at an 80% confluence level (**Supplementary Figure 1B**). To characterize the immunophenotype of the isolated cells, HIMFs were seeded onto chamber slides (#30108, SPL Life Sciences, Korea) and fixed for immunostaining. The cells were stained with primary antibodies against α-SMA (A2547, Sigma), vimentin (ab92547, Abcam), and desmin (ab15200, Abcam). After mounting with Fluoroshield Mounting Medium containing DAPI (ab104139, Abcam), fluorescence images were acquired using a confocal microscope. HIMFs were identified based on positive staining for α-SMA and vimentin, and negative staining for desmin. For the negative control, only DAPI staining was performed (**Supplementary Figure 1C**).

### 2.5 Three-step experimental design investigating the impact of damaged enteroid monolayers on subepithelial myofibroblasts

**Figure 1:**
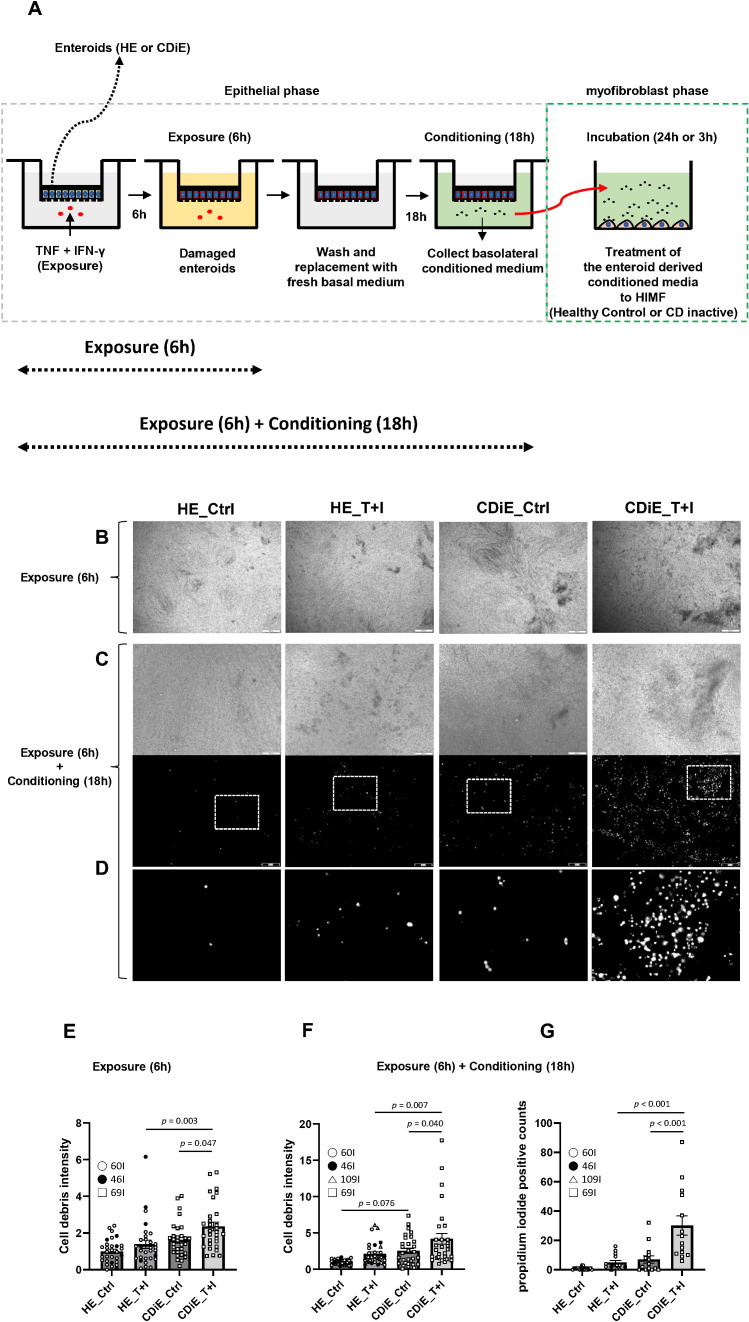
TNF and IFN- γ induce cell death in ileal enteroid monolayers. (A) Schematic illustration of the experimental design, investigating the impact of TNF and IFN-γ on ileal enteroid monolayers: (i) TNF and IFN-γ exposure for 6 hours on lower chamber in healthy and CD inactive enteroids (HE_T+I and CDiE_T+I). Control enteroids (HE_Ctrl, CDiE_Ctrl) were not exposed to the cytokines. (ii) after the 6-hour exposure, all the media for all groups were removed out, washed with PBS, and replaced by fresh basal medium. Additional 18-hour conditioning was done (no presence of T+I). For the conditioning period, nothing else was added to the conditioning media for all groups. This procedure corresponds to the epithelial phase indicated by the gray dotted box in **Figure 1A**. **(B)** Phase contrast images of ileal enteroid monolayers after 6-hour exposure of T+I. The images show both unexposed and exposed monolayers. Conditions represented are: HE_Ctrl (healthy enteroid control, no exposure), HE_T+I (healthy enteroid with TNF and IFN-γ exposure), CDiE_Ctrl (CD inactive enteroid control, no exposure), and CDiE_T+I (CD inactive enteroid with TNF and IFN-γ exposure). *Scale bar: 200 µm*. **(C, D)** Following 6-hour T+I exposure plus an additional 18-hour conditioning, phase contrast **(C)** and propidium iodide fluorescence images **(D)** of ileal enteroid monolayers were captured for the above four conditions (*Scale bar: 200 µm*). Images in **Figures 1B–D** are representative of similar results in 3–5 independent experiments. Enlarged views of the representative regions (indicated by white boxes) are displayed below each corresponding propidium iodide image. **(E)** Cell debris intensity post 6-hour exposure was determined using ImageJ software in 30 separate, same sized surface areas (16,480 µm^2^) from 4 independent experiments. **(F)** Cell debris intensity following 6-hour exposure plus 18-hour conditioning was determined using ImageJ software in 30 separate, same sized surface areas (16,480 µm^2^) from 5 independent experiments. **(G)** Propidium Iodide (PI)-positive cell counts were determined using ImageJ software in 14 separate, same sized surface areas (16,480 µm^2^) from 3 independent experiments. **(E–G)** Results presented as means ± standard error of the mean (SEM), with statistical analysis performed using ANOVA followed by Tukey's *post-hoc* testing. Enteroid lines used for phase contrast images or PI staining include HE (n=3; 60I, 46I, 109I) and CDiE (n=1; 69I). Individual data points are displayed as unique symbols superimposed on bar graphs to identify each independent donor line and ensure data transparency.

To elucidate the effects of damaged enteroid monolayers on subepithelial myofibroblasts, a three-step experimental approach was used (**Figure 1A**). In the first step, the organoid monolayers were exposed to TNF (100 ng/mL) and IFN-γ (50 ng/mL) for 6 hours to induce a Th1-like response characteristic of CD. During this initial stimulation, A83–01 and SB202190 were included in the medium to maintain the structural integrity and undifferentiated state of the monolayers against acute cytokine-induced stress. For the second step, all media were removed, followed by a PBS wash. The medium was then replaced with cytokine-free expansion medium from which A83–01 and SB202190 were strictly excluded (Foulke-Abel et al., 2020). This removal ensured that the profibrotic signaling pathways, particularly the TGF-β pathway, remained uninhibited, allowing for an accurate evaluation of the epithelial-mesenchymal interaction. After an 18-hour conditioning period, the basolateral conditioned media were collected. In the final step, these media were used to incubate HIMF for 3 or 24 hours (**Figure 1A**).

### 2.6 Immunofluorescence staining

Organoid monolayers on Transwell filters were fixed using 4% paraformaldehyde for 30 minutes at room temperature, washed three times for 5 minutes with Tris-buffered saline (TBS), permeabilized with 0.2~0.3% Triton X-100 in TBS for 30 minutes, and blocked using TBS containing 15% fetal bovine serum (FBS) for 30 minutes. The cells were then immunostained with the following primary antibodies, typically overnight at 4˚C. Mouse monoclonal anti-occludin antibodies (1:100, Invitrogen, USA; Cat#SAB4200489) and rabbit polyclonal anti-Ki67 (1:50, Santa Cruz, USA; Cat#SC-15402). All primary antibody dilutions were made in blocking solution or TBS. The monolayers were washed three times with TBS and incubated with Alexa Fluor-conjugated secondary antibodies (1:100), Hoechst 405 (1:200, Vector Laboratories, USA) for nuclear/DNA labeling, and fluorescence-conjugated phalloidin 633 for filamentous actin (F-actin) staining (1:100, Life Technologies, USA; Cat#A22284) diluted in TBS, for 1 hour at room temperature. Secondary antibodies included Goat anti-Rabbit-Alexa-568 and Goat anti-Mouse-Alexa-488 (Molecular Probes/Invitrogen, USA). The filters were cut away from the insert, then immersed in gel mount (ProLong Gold, Vector Laboratories, USA) and mounted onto glass slides.

### 2.7 Confocal microscopy

Confocal imaging was executed at the Fluorescence Imaging Core of the Hopkins Basic Research Digestive Disease Development Center using a Zeiss LSM-510 META laser scanning confocal microscope (Zeiss, Germany). All images were acquired as Z-stacks with a 20X oil objective. To ensure consistency across experimental groups, the apical junctional complex was identified by the peak intensity of occludin, and a representative single Z-plane (typically at a depth of -3.09 µm) was selected for analysis. While settings were optimized for qualitative assessment, strictly consistent parameters were maintained for all quantitative comparisons. Furthermore, orthogonal views (XZ and YZ sections) were reconstructed from the Z-stacks to verify vertical focus alignment and visualize the perijunctional F-actin belt.

### 2.8 Cell debris quantification

Cell debris intensity ratio was determined using ImageJ software in 30 separate, same sized surface areas (16,480 µm^2^) from at least 4 independent experiments. In phase contrast images, cell debris was defined as discrete, irregularly shaped extracellular particles with sharp edges and strong contrast relative to the background. Only particles clearly distinguishable from the intact epithelial monolayer were included. Diffuse haze, shadows, and refractile background artifacts were excluded to avoid false-positive quantification. To adjust the background density, the mean intensity of 2 distinct same sized background areas (16,480 µm^2^) was subtracted from the cell debris intensity of each image.

### 2.9 Propidium iodide-positive cell quantification

PI-positive cells were defined as sharply bordered, well-circumscribed nuclear signals with high fluorescence intensity. Only distinct nuclear staining patterns were included in the analysis. Diffuse haze, blurred fluorescence, or cloud-like signals were excluded to avoid counting background artifacts or non-specific staining. Propidium Iodide (PI)-positive cell counts were determined using ImageJ software in 14 separate, same sized surface areas (16,480 µm^2^) from 3 independent experiments.

### 2.10 Other quantitative analyses

Quantification of Ki67 expression in enteroid monolayers was determined by normalizing against the Hoechst positive cell counts using ImageJ software. Measurements were taken across 45 distinct same sized surface areas (8,144 µm^2^) from 3 independent experiments. The fluorescence intensity ratios of occludin and F-actin to Hoechst were quantitatively analyzed using ImageJ software. For junctional protein quantification (occludin), local line-quantification was performed specifically along the tight junctions to exclude non-specific cytoplasmic signals. Measurements were taken across 24 distinct same sized surface areas (602.5 µm^2^ for images taken with zoom factor 3.71, 610 µm^2^ for images taken with zoom factor 1.0) from 3 independent experiments.

### 2.11 Quantitative real-time polymerase chain reaction

Total RNA was extracted using the PureLink RNA Mini Kit (Life Technologies) according to the manufacturer’s protocol. Complementary DNA was synthesized from 1 µg of RNA using SuperScript VILO Master Mix (Life Technologies). Quantitative real-time polymerase chain reaction (qRT-PCR) was performed using Power SYBR Green Master Mix (Life Technologies) on a QuantStudio 12K Flex real-time PCR system (Applied Biosystems, Foster City, CA). Each sample was run in triplicate, and 5 ng RNA-equivalent complementary DNA was used for each reaction. The sequences of gene-specific primers are listed in **Table 2**. The relative fold changes in mRNA levels of proinflammatory and profibrotic genes in HIMF were determined using the 2^-ΔΔCT^ method with human *GAPDH* (Glyceraldehyde 3-Phosphate Dehydrogenase) RNA simultaneously studied and used as the internal control for normalization.

**Table 2:** Gene-specific primers for qRT-PCR.

Gene	Forward (5’-3’)	Reverse (5’-3’)
*COL1A1*	GAGGGCCAAGACGAAGACATC	CAGATCACGTCATCGCACAAC
*ACTA2*	AAAAGACAGCTACGTGGGTGA	GCCATGTTCTATCGGGTACTTC
*TGFB*	CGACTACTACGCCAAGGAGG	CGGAGCTCTGATGTGTTGAA
*SRF*	CGAGATGGAGATCGGTATGGT	GGGTCTTCTTACCCGGCTTG
*SMAD7*	TTCCTCCGCTGAAACAGGG	CCTCCCAGTATGCCACCAC
*IL1B*	ATGATGGCTTATTACAGTGGCAA	GTCGGAGATTCGTAGCTGGA
*IL6*	CCACTCACCTCTTCAGAACG	CATCTTTGGAAGGTTCAGGTTG
*MCP1*	CAGCCAGATGCAATCAATGCC	TGGAATCCTGAACCCACTTCT
*IKBA*	CTCCGAGACTTTCGAGGAAATAC	GCCATTGTAGTTGGTAGCCTTCA
*GAPDH*	AGGTCGGAGTCAACGGATTTGG	ACAGTCTTCTGGGTGGCAGTGATG

*COL1A1*, Collagen Type I, Alpha 1; *ACTA2*, α-Smooth Muscle Actin; *TGFB*, Transforming Growth Factor Beta 1; *SRF*, Serum Response Factor;.

*SMAD7*, SMAD7; *IL1B*, Interleukin-1 Beta; *IL6*, Interleukin-6; *MCP1*, Monocyte Chemoattractant Protein-1; *IKBA*, IĸBα; *GAPDH*, Glyceraldehyde 3-Phosphate Dehydrogenase.

### 2.12 Statistical analysis

Quantitative data were derived from human ileal enteroids established from four healthy subjects (**Table 1**) and represented using Superplots, where independent experimental data points are superimposed on bar graphs, with each donor line identified by a unique symbol. The specific number of donor lines used for each experiment is indicated in the corresponding figure legends and **Supplementary Table 1**. All error bars indicate the Standard Error of the Mean (SEM) from a minimum of three independent experiments, except where noted (**Supplementary Figure 2**). Statistical analyses were performed using ANOVA followed by Tukey’s *post-hoc* test, with a P-value < 0.05 considered statistically significant. All analyses were conducted using GraphPad Prism 11.0.

## 3 Results

### 3.1 TNF and IFN-γ induce cell death in CD enteroids

In **Figure 1A**, the experimental design to elucidate the influence of TNF and IFN-γ on ileal enteroids is shown. Enteroids from healthy subjects (HE) and inactive CD enteroids (CDiE) were either not otherwise treated or exposed to TNF (short notation- T) and IFN-γ (I) for 6 hours (HE_T+I, CDiE_T+I). Six hours of T+I exposure significantly increased cell debris intensity in CDiE (*p* = 0.047), as demonstrated by the representative phase-contrast images (**Figure 1B**) and the corresponding quantification (**Figure 1E**). Under baseline conditions, CDiE had a slightly but not significantly larger amount of cell debris than HE (**Figures 1B, E**). However, cell debris intensity was significantly higher in CDiE_T+I than HE_T+I (*p* = 0.003). We also determined if enteroids made from CD active enteroids (CDaE) behaved similarly to CDiE using the same experimental design as in **Figure 1A**. The results were similar, except that baseline CDaE had significantly (*p* < 0.001) higher cell debris intensity than HE. Six hours of T+I exposure significantly increased cell debris intensity in CDaE but not in HE (**Supplementary Figures 2A, D**).

As shown in **Figure 1A**, after the 6-hour T+I exposure, all the media for all groups (HE_Ctrl, HE_T+I, CDiE_Ctrl, CDiE_T+I) were removed, washed with PBS, replaced by fresh basal medium, and subsequent 18-hour conditioning was done. Nothing else was added to the fresh basal medium for the 18-hour conditioning period for all groups. Six hours of T+I exposure plus an additional 18-hour conditioning did not significantly alter cell debris intensity in HE, but significantly increased it in CDiE (*p* = 0.040), as shown in **Figures 1C, F**. Under baseline conditions, CDiE has a slightly but not significantly (*p* = 0.076) larger amount of cell debris than HE. However, cell debris intensity was significantly higher in CDiE_T+I than HE_T+I (*p* = 0.007).

We also investigated whether CDaE exhibited similar behavior to CDiE using the same experimental design of **Figure 1A**. As shown in **Supplementary Figures 2B, E**, six hours of T+I exposure followed by an additional 18-hour conditioning resulted in no significant change in cell debris intensity in HE, whereas a significant increase in cell debris intensity was observed in CDaE. Under baseline conditions, unlike CDiE, cell debris intensity was significantly higher in CDaE compared to HE (*p* = 0.009). Additionally, under T+I exposure, CDaE continued to exhibit significantly elevated debris intensity than HE. These findings show that the pattern of changes in cell debris intensity following T+I exposure remains consistent between the initial 6-hour exposure and the subsequent 18-hour conditioning in CDaE, mirroring the results observed in CDiE.

Propidium iodide (PI) staining of the enteroid monolayer was used to quantify the extent of enteroid cell death. Under baseline conditions, there was no significant difference in PI positive cell counts in HE, CDiE and CDaE; six hours of T+I exposure plus additional 18-hour conditioning had no effect on PI-positive counts in HE but caused a significant increase in PI-positive counts in CDiE and CDaE, as shown in **Figures 1D, G** and **Supplementary Figures 2C, F**.

In summary, T+I exposure induces epithelial damage (manifested as increased cell debris and cell death) in both CDiE and CDaE, with a similar effect in both, while having no effect on HE. Notably, upon T+I exposure, both CDiE and CDaE show significantly higher cell debris intensity and PI-positive counts compared to HE. This finding suggests that the ileal enteroids from CD (CDiE and CDaE) are more susceptible to damage by T+I compared to HE. Interestingly, under baseline conditions, PI-positive cell counts did not differ between HE, CDiE, and CDaE, likely reflecting the fact that enteroids are typically derived from relatively preserved epithelial regions rather than severely damaged mucosa in active CD. However, while cell debris intensity was comparable between HE and CDiE, it was significantly elevated in CDaE compared to HE. These findings suggest that CDiE retains epithelial integrity comparable to HE at baseline, whereas CDaE may be more susceptible to epithelial injury than CDiE.

### 3.2 TNF and IFN-γ reduce epithelial proliferation in ileal enteroids

TNF has been documented to suppress the number of intestinal stem cells and the proliferation of enteroids (Saito et al., 2021; Lee et al., 2022). Concurrently, elevated levels of both TNF and IFN-γ in the mucosa of IBD patients have been implicated in exacerbating intestinal barrier dysfunction and increasing intestinal permeability. This is primarily achieved through the perturbation of tight junctions, including altered expression of tight junction proteins and is facilitated by the cytoskeletal reorganization of IECs (Bruewer et al., 2003; Yoo and Donowitz, 2019). Consequently, we determined if changes in enteroid proliferation and barrier dysfunction were induced by TNF and IFN-γ exposure. Measurements were made of cytokine induced changes in enteroid cell proliferation by measuring Ki67 immunofluorescence, and the expression levels of both a tight junctional protein (occludin) and a cytoskeletal protein (filamentous actin; F-actin).

**Figure 2:**
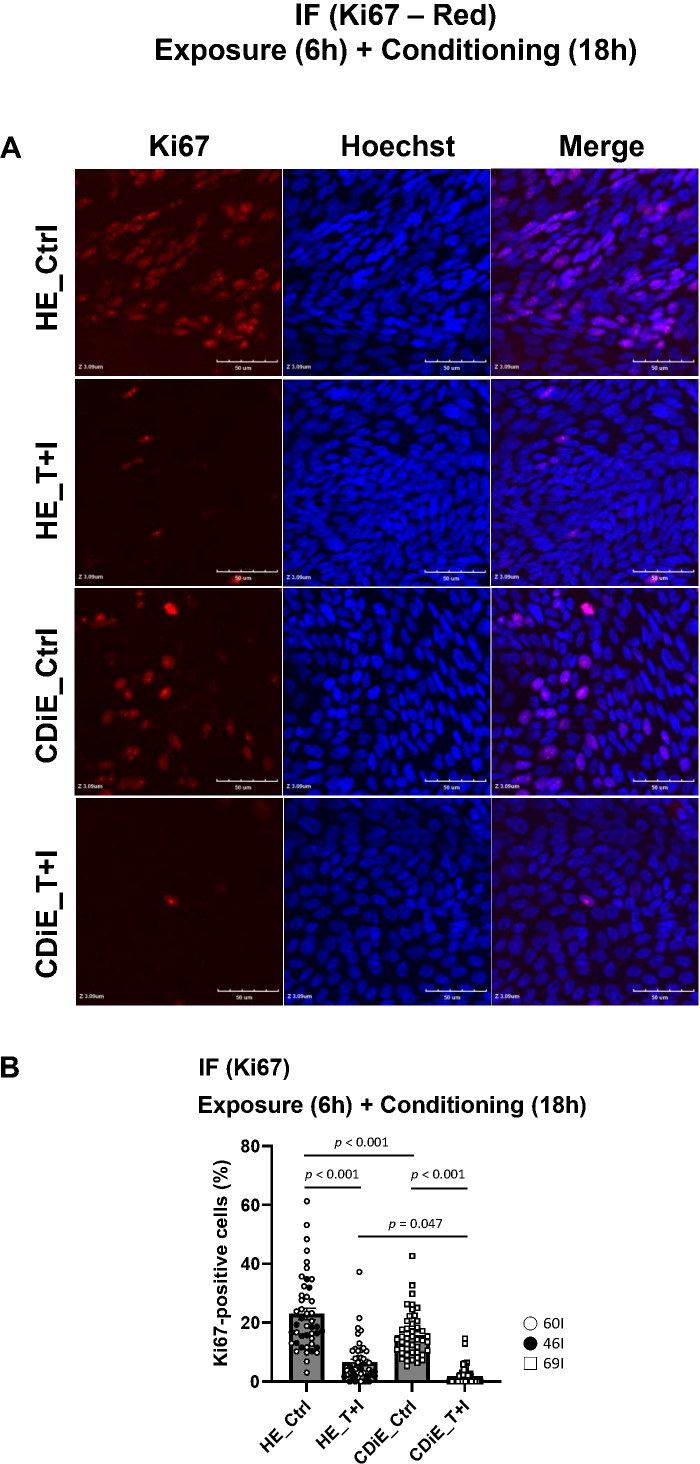
Effect of TNF and IFN-γ on epithelial proliferation in ileal enteroid monolayers. **(A)** After 6-hour exposure with T+I, followed by an 18-hour conditioning, Ki67 nuclear staining was carried out in ileal enteroid monolayers. Visualization was achieved with Ki67 (red) and Hoechst (blue) expression. Representative images for the following conditions are displayed: HE_Ctrl, HE_T+I, CDiE_Ctrl, and CDiE_T+I. Confocal X-Y images. All the X-Y images were taken at same level (Z 3.09 µm). *Scale bar: 50 µm* **(B)** Quantification of Ki67 expression in enteroid monolayers: The percentage of epithelial cells with positive nuclear Ki67 staining was calculated by normalizing against the total cell count, as determined using ImageJ. Measurements were taken across 45 distinct same sized surface areas (8,144 µm^2^) from 3 independent experiments. The results are presented as means ± SEM, and statistical significance was determined using ANOVA followed by a Tukey's *post-hoc* test. For immunofluorescence staining, the enteroid lines used were HE (n=2; 60I, 46I) and CDiE (n=1; 69I).

Under baseline conditions, the percentage of Ki67 positive cells was lower in CDiE than HE; following exposure to T+I for 6 hours plus additional 18-hour conditioning (**Figure 1A**), a significant reduction in the percentage of Ki67-positive cells was observed in both HE and CDiE, as shown in **Figures 2A, B**. Furthermore, CDiE_T+I exhibited a notably lower percentage of Ki67-positive cells compared to HE_T+I. Thus, CDiE exhibit reduced proliferation compared to HE under both baseline conditions and after exposure to T+I. Unlike cell death, cell proliferation was significantly reduced by T+I in both HE and CDiE. Notably, under baseline conditions, CDiE showed reduced proliferation compared to HE, despite no difference in cell death. This dissociation highlights a key strength of the CDiE model: epithelial integrity is maintained, while impaired proliferative capacity is already present, suggesting early epithelial vulnerability that may contribute to initiation of inflammation or fibrosis.

### 3.3 TNF and IFN-γ reduce the expression of occludin and f-actin in ileal enteroids

**Figure 3:**
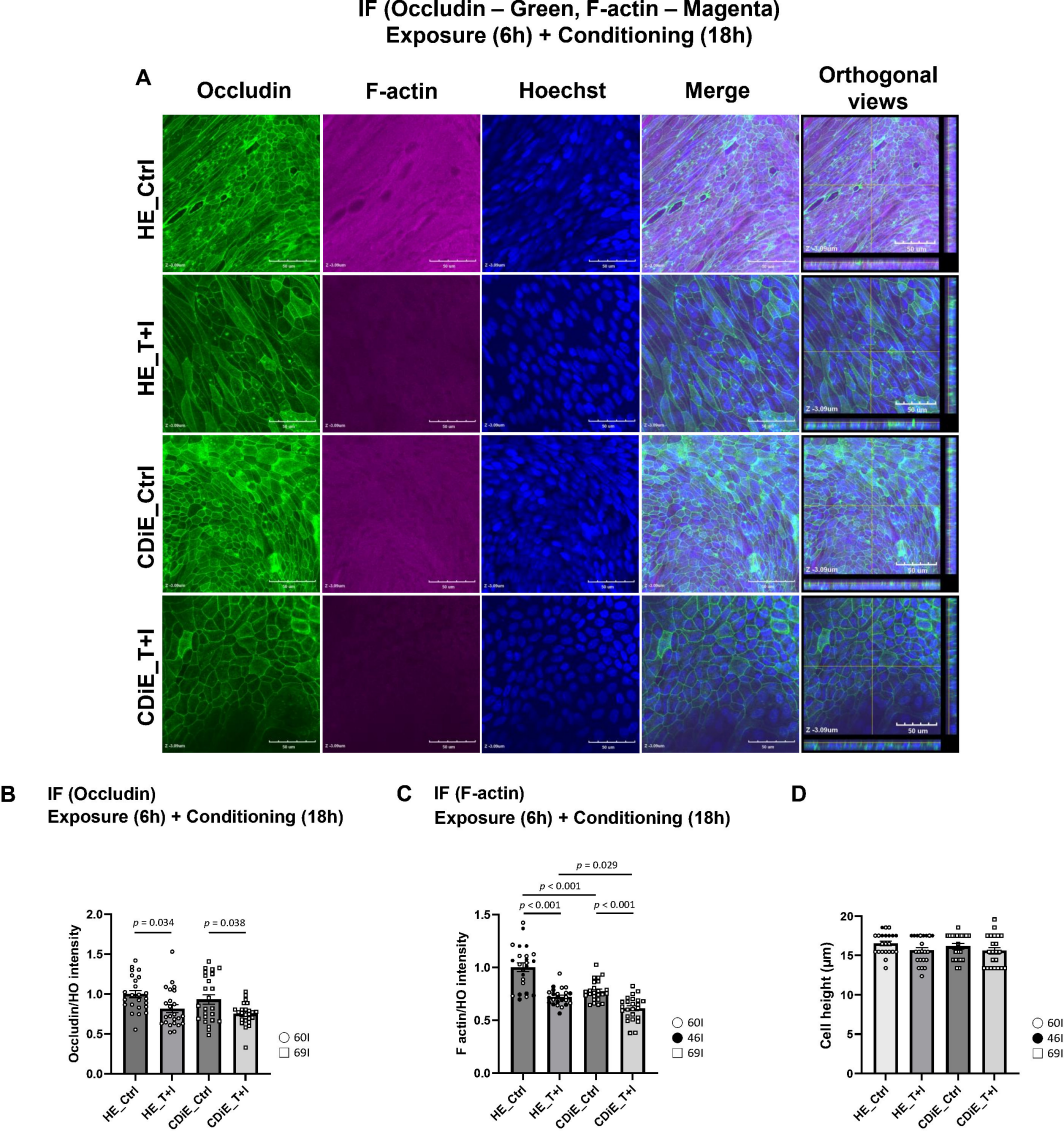
Effect of TNF and IFN-γ on the expression of occludin and F-actin in ileal enteroid monolayers. **(A)** Representative confocal images: After 6-hour exposure to TNF and IFN**-γ** (T+I) followed by 18-hour conditioning, the expression of occludin (green) and F-actin (magenta) was visualized by immunofluorescence staining; nuclei were counterstained with Hoechst (blue). To ensure standardized comparison, all XY-planes were acquired at the same physiological level (standardized at Z -3.09 µm based on peak occludin intensity). The rightmost panels display orthogonal views (XZ and YZ sections) reconstructed from Z-stacks to verify vertical focus alignment and the perijunctional actin belt. *Scale bar: 50 µm*. **(B)** Quantitative analysis of occludin: Fluorescence intensities were determined via local line-quantification along the tight junctions to exclude non-specific cytoplasmic signals. The fluorescence intensity ratio of occludin to Hoechst was quantified and plotted using ImageJ software. Measurements were taken across 24 distinct same sized surface areas (602.5 µm^2^ for images taken with zoom factor 3.71, 610 µm^2^ for images taken with zoom factor 1.0) from 3 independent experiments. The results are presented as means ± SEM, and statistical significance was determined using ANOVA followed by a Tukey's *post-hoc* test. For immunofluorescence staining, the enteroid lines used were HE (n=1; 60I) and CDiE (n=1; 69I). **(C)** Quantitative analysis of F-actin: The fluorescence intensity ratio of F-actin to Hoechst was quantified and plotted using ImageJ software: Measurements were taken across 24 distinct same sized surface areas (602.5 µm^2^ for images taken with zoom factor 3.71, 610 µm^2^ for images taken with zoom factor 1.0) from 3 independent experiments. The results are presented as means ± SEM, and statistical significance was determined using ANOVA followed by a Tukey's *post-hoc* test. For immunofluorescence staining, the enteroid lines used were HE (n=2; 60I, 46I) and CDiE (n=1; 69I). **(D)** Quantitative analysis of cell height: To investigate potential three-dimensional morphological changes, cell height was measured from orthogonal images (XZ sections) of monolayers dual-stained for F-actin and Hoechst. Measurements were obtained from 22 distinct locations per group across five independent experiments. The results are presented as means ± SEM, and statistical significance was determined using ANOVA followed by a Tukey's *post-hoc* test. For immunofluorescence staining, the enteroid lines used were HE (n=2; 60I, 46I) and CDiE (n=1; 69I).

Additional studies were conducted to investigate the effects T+I exposure on occludin and F-actin. Following a 6-hour T+I exposure and an additional 18-hour conditioning period, occludin expression was significantly reduced in both HE and CDiE, as shown by immunofluorescence staining (**Figure 3A**) and quantitative analysis (**Figure 3B**). Notably, the occludin/Hoechst intensity ratio in CDiE did not differ significantly from that in HE under either untreated conditions or after T+I exposure. Similarly, a significant reduction in the normalized intensity of F-actin was observed in both HE and CDiE (**Figure 3C**) after T+I exposure. The expression of F-actin in CDiE was significantly lower than in HE, independent of T+I exposure.

To determine if the visually apparent expansion of apical areas in T+I-treated monolayers reflected a genuine change in cell shape, we performed XZ-section analysis to measure cell height (**Figure 3D**). Quantitative measurements revealed no significant change in cell height in either group, indicating that the morphological remodeling primarily involves lateral cell flattening rather than vertical shrinkage.

Overall, these results indicate that exposure to TNF and IFN-γ reduces the expression of occludin and F-actin in ileal enteroids from both healthy controls and inactive CD patients. In contrast to occludin, which showed no difference between HE and CDiE under baseline conditions, F-actin expression was lower in CDiE than in HE. This finding suggests that, despite preserved tight junction protein levels, CDiE may exhibit cytoskeletal instability at baseline, potentially predisposing the epithelium to increased vulnerability under inflammatory stress.

### 3.4 TNF and IFN-γ reduce TEER in CD-derived but not healthy enteroids

**Figure 4:**
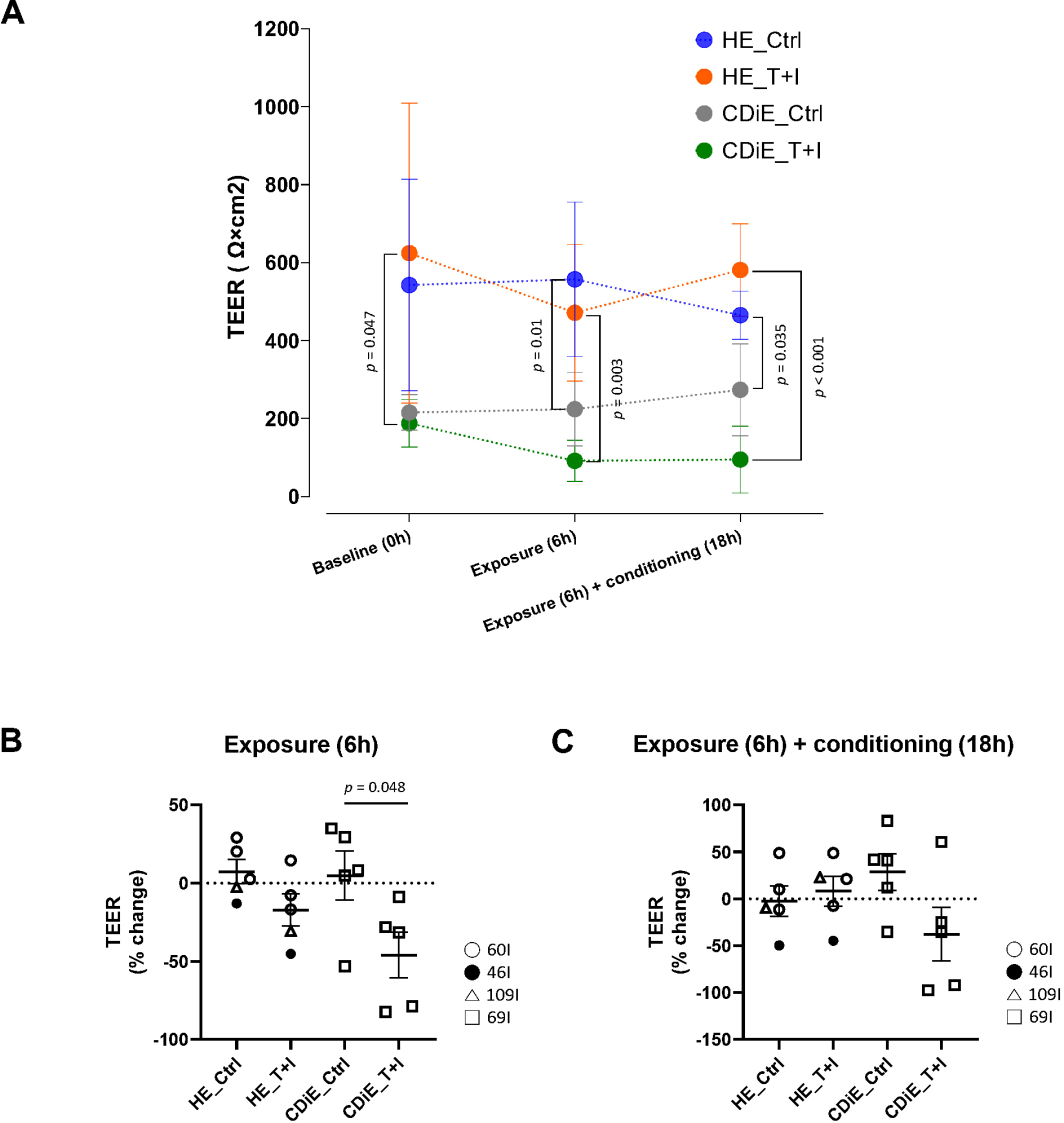
Effect of TNF and IFN-γ on epithelial permeability in ileal enteroid monolayers. **(A)** Transepithelial electrical resistance (TEER) of ileal enteroid monolayers were measured at 3 different time points – baseline, after 6-hour exposure of T+I, and after 6-hour exposure of T+I plus 18-hour conditioning for all groups (HE_Ctrl, HE_T+I, CDiE_Ctrl, and CDiE_T+I). **(B)** The percentage changes in TEER between two time points (baseline and after 6-hour exposure) were calculated and plotted for all groups. **(C)** The percentage changes in TEER between two time points (baseline and after 6-hour exposure of T+I plus 18-hour conditioning) were calculated and plotted for all groups. **(A-C)** Data are from 5 independent experiments. For TEER measurement, the enteroid lines used were HE (n=3; 60I, 46I, 109I) and CDiE (n=1; 69I). Error bars represent the SEM, and statistical significance was determined using ANOVA followed by Tukey’s *post-hoc* test.

We hypothesized that epithelial changes following T+I exposure—including increased cell debris, cell death, and reduced proliferation, along with decreased expression of occludin and F-actin—may contribute to increased epithelial permeability in CDiE. To evaluate the effect of inflammation on a parameter of epithelial permeability, we investigated the effect of a 6-hour cytokine exposure, with and without an additional 18-hour conditioning (**Figure 1A**), on the TEER of enteroids. **Figure 4A** shows the TEER value at each condition of baseline (0 hours, before cytokine exposure), exposure (6 hours), and exposure (6 hours) + conditioning (18 hours) for the four enteroid groups (HE_Ctrl, HE_T+I, CDiE_Ctrl, CDiE_T+I). TEER in HE did not significantly change when observed for 6 hours and 24 hours (including an 18-hour conditioning period). At baseline, after 6 hours, and after 6-hours + additional 18-hour conditioning, otherwise untreated CDiE had a lower TEER compared to HE and did not change significantly over time (**Figure 4A**). HE did not undergo significant change in TEER after T+I exposure either for 6 hours or 6-hour exposure + additional 18-hour conditioning. In contrast, CDiE exposed to T+I showed approximately 50% reduction in TEER at both 6 hours and after an additional 18-hour conditioning period, although the latter did not reach statistical significance. In **Supplementary Figure 3A**, we investigated the TEER values for CDaE using the same experimental design as in **Figure 4**. Under all three conditions (baseline, 6 hours of exposure, and 6 hours of exposure followed by 18 hours of conditioning), CDaE had a lower TEER than HE, although this was not statistically significant.

Analysis of TEER changes, expressed as percent change from baseline to 6 hours of T+I exposure (**Figure 4B** and **Supplementary Figure 3B**) and from baseline to 6 hours plus an additional 18-hour conditioning period (**Figure 4C** and **Supplementary Figure 3C**), further demonstrated that TEER remained stable in HE over time and with T+I exposure, whereas both CDiE and CDaE had lower baseline TEER compared to HE and exhibited further reductions following T+I exposure. These results which did not reach statistical significance suggest that one parameter of epithelial permeability, TEER, is lower in ileal enteroid monolayers derived from CD patients (CDiE, CDaE) compared to HE, under both basal and damaged conditions and only the CD enteroids were sensitive to T+I by responding to their exposure with a reduced TEER.

### 3.5 Cytokine-exposed ileal enteroid conditioned media triggers proinflammatory genes while suppressing profibrotic genes in HIMF-HC and HIMF-CD

**Figure 5:**
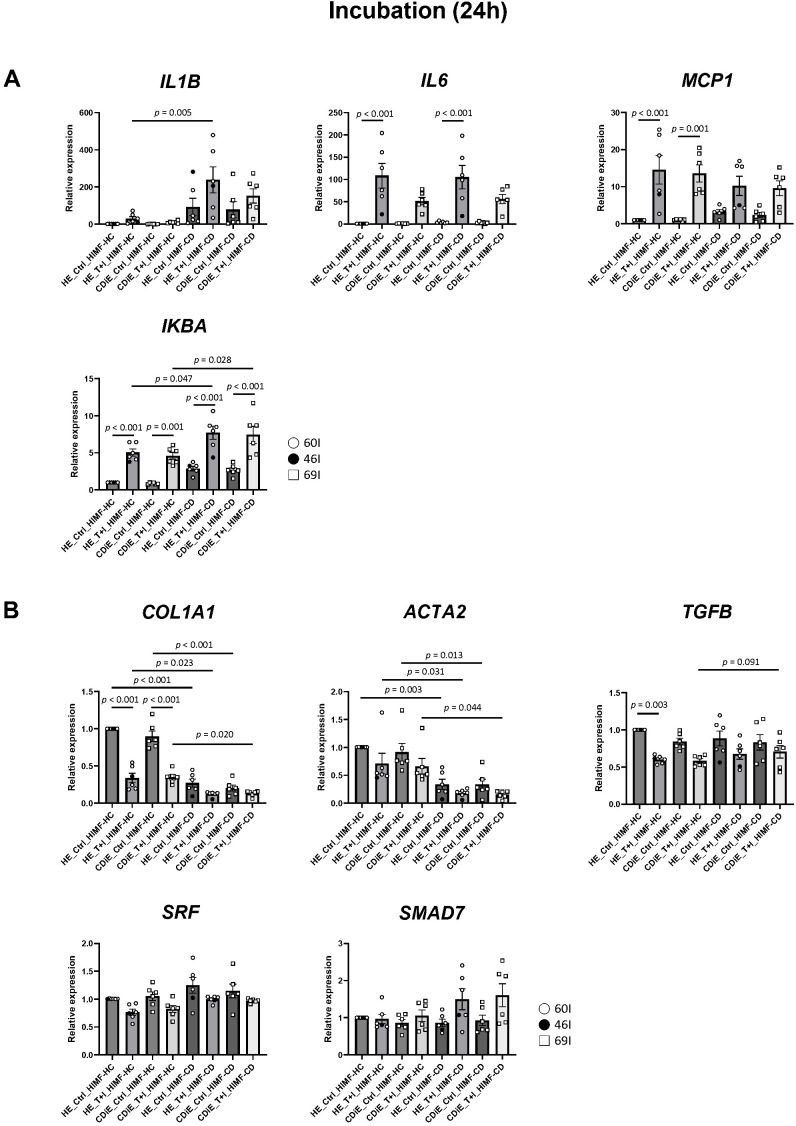
Effects of cytokine-exposed enteroid-derived conditioned media on proinflammatory and profibrotic gene transcription in human ileal myofibroblasts. After 6 hours of T+I exposure and an additional 18-hour conditioning, the resulting basolateral conditioned media were applied to human ileal myofibroblasts for 24 hours (see **Figure 1A**). This procedure corresponds to the myofibroblast phase indicated by the green dotted box in **Figure 1A**. We analyzed differential gene expression profiles in two types of HIMF: those derived from healthy mucosa of healthy subjects (HIMF-HC) and those from inactive ileal mucosa of Crohn’s disease patients (HIMF-CD). Four distinct enteroid-derived conditioned medium conditions were examined: HE_Ctrl, HE_T+I, CDiE_Ctrl, and CDiE_T+I. **(A)** mRNA expression of proinflammatory genes (*IL1B*, *IL6*, *MCP1*, and *IKBA*) in HIMF-HC and HIMF-CD after 24-hour incubation with enteroid-derived conditioned media. **(B)** mRNA expression of profibrotic genes (*COL1A1*, *ACTA2*, *TGFB*, and *SRF*) and the anti-fibrotic gene *SMAD7* after 24-hour incubation. Data were normalized to *GAPDH*. The results are presented as means ± SEM, and statistical significance was determined using ANOVA followed by a Tukey's *post-hoc* test for 6 independent experiments. For quantitative PCR, the enteroid lines used were HE (n=2; 60I, 46I) and CDiE (n=1; 69I).

To investigate the impact of conditioned media from cytokine-exposed ileal enteroids on proinflammatory and profibrotic gene expression in subepithelial myofibroblasts, we treated subepithelial myofibroblasts derived from healthy ileum (HIMF-HC) and CD inactive ileum (HIMF-CD) with conditioned medium from enteroids (HE and CDiE), with or without 6 hours of T+I exposure followed by 18 hours of conditioning. These incubations were conducted for either 24 hours (**Figure 5**) or 3 hours (**Supplementary Figure 4**), as illustrated in the experimental design shown in **Figure 1A**.

Incubation of HIMF-HC with conditioned medium from cytokine-exposed enteroids (HE_T+I and CDiE_T+I) for 24 hours significantly increased, or tended to increase, the mRNA expression levels of proinflammatory genes, including *IL6*, *MCP1*, and *IKBA*, compared to the control conditioned medium from cytokine-unexposed enteroids (HE_Ctrl and CDiE_Ctrl); *IL1B* minimally increased with the changes not reaching statistical significance. HIMF-CD showed similar results to HIMF-HC, although with a significant increase in *IL6* and *IKBA* (**Figure 5A**).

Treatment with conditioned medium from cytokine-exposed enteroids (HE_T+I and CDiE_T+I) for 3 hours produced similar results in both HIMF-HC and HIMF-CD regarding the mRNA expression levels of *IL1B*, *MCP1*, and *IKBA*, although with a significant increase in *MCP1* and *IKBA* in response to CDiE_T+I, as shown in **Supplementary Figure 4**. In contrast, in studies which compared the effects of 3 hour and 24 hour-exposure to cytokine-unexposed enteroid-derived conditioned medium (HE and CDiE) and basal medium (BM) on HIMF-HC and HIMF-CD (**Supplementary Figures 5A, B**), mRNA levels of *IL1B*, *IL6*, *MCP1*, and *IKBA* were slightly but not significantly higher in HIMF-CD compared to HIMF-HC. The only significant difference was higher *IKBA* in HIMF-CD after 3 hour-exposure to otherwise untreated CDiE conditioned media or BM.

We evaluated the impact of conditioned media from cytokine-exposed enteroids on the expression of profibrotic genes in subepithelial myofibroblasts (**Figure 5B**). Notably, the results for profibrotic genes contrasted with those for proinflammatory genes. Incubation of HIMF-HC with conditioned medium from cytokine-exposed enteroids (HE_T+I and CDiE_T+I) for 24 hours significantly decreased, or tended to decrease, mRNA expression levels of profibrotic genes, including *COL1A1*, *ACTA2*, *TGFB*, and *SRF*, compared to control conditioned medium from cytokine-unexposed enteroids (HE_Ctrl and CDiE_Ctrl).

Under the same experimental conditions, HIMF-CD showed similar results to HIMF-HC with reduced profibrotic genes in response to conditioned media from T+I exposure to HE and CDiE enteroids (**Figure 5B**). However, for *COL1A1* and *ACTA2*, but not *TGFB* or *SRF*, HIMF-CD exhibited lower levels of mRNA expression compared to HIMF-HC following incubation with HE- or CDiE-conditioned media, both with and without T+I exposure. These results were consistent with supplementary data (**Supplementary Figure 5C**), which showed that after 24 hours of incubation with the same medium conditions (BM, HE- and CDiE-conditioned medium), *COL1A1* and *ACTA2* mRNA levels were significantly lower in HIMF-CD compared to HIMF-HC.

These changes in profibrotic genes could potentially be attributed to alterations in the TGF-β/Smad7 signaling pathway (Lawrance et al., 2001; Monteleone et al., 2004; Di Sabatino et al., 2009). In contrast to the response of profibrotic genes, incubation of HIMF-HC and HIMF-CD with conditioned medium from cytokine-exposed enteroids (HE_T+I and CDiE_T+I) for 24 hours did not significantly affect the expression of antifibrotic gene *SMAD7* (**Figure 5B**). In summary, these results suggest that conditioned media from TNF and IFN-γ-exposed healthy and CD inactive ileal enteroids activate proinflammatory genes while suppressing profibrotic genes in both HIMF-HC and HIMF-CD. Under the same conditions, HIMF-CD tended to exhibit higher expression of proinflammatory genes (*IL1B*, *IL6*, *MCP1*, and *IKBA*) and lower expression of profibrotic genes (*COL1A1* and *ACTA2*) compared to HIMF-HC.

## 4 Discussion

Approximately half of CD patients with ileal disease undergo intestinal resection within 10 years of diagnosis (Frolkis et al., 2013, 2014). Stricturing complications account for 40–70% of these surgeries (Rieder et al., 2013; Sleiman et al., 2021), and recurrence, particularly at ileocolonic anastomotic sites, is common (Rutgeerts et al., 1990). About 35% of patients require repeat surgery within 10 years (Frolkis et al., 2014). However, whether controlling ileal inflammation post-surgery effectively prevents stricture recurrence remains unclear.

This study modeled the immediate post-ileal resection state in Crohn’s disease (CD) using human ileal enteroids and myofibroblasts derived from healthy and inactive CD ileum. The goal was to determine whether myofibroblasts from inactive CD ileum produce proinflammatory and profibrotic cytokines that could initiate post-resection fibrosis. Myofibroblast cytokine responses were evaluated under baseline conditions and following exposure to conditioned media from healthy and inactive CD enteroids, both at baseline and after epithelial damage induced by TNF plus IFN-γ treatment (6 hours exposure, followed by an 18-hour washout). Thus, this model assessed both epithelial and mesenchymal contributions to fibrosis initiation.

Ileal enteroids derived from inactive CD exhibited notable differences from healthy enteroids under baseline conditions, including reduced proliferation, decreased F-actin expression, slightly increased cell death, and reduced TEER. These abnormalities were even more pronounced in enteroids from active CD areas. Upon inflammatory stimulation with TNF plus IFN-γ, healthy enteroids showed no change in cell death, whereas CD enteroids (particularly from active regions) exhibited increased cell death. Inflammation also similarly decreased proliferation (**Figure 2**) and reduced occludin and F-actin expression (**Figure 3**) in both healthy and inactive CD enteroids. Notably, inflammation did not affect TEER in healthy enteroids but reduced TEER (~50%) in inactive CD enteroids (**Figure 4A**). In summary, these observations demonstrate that CD-derived epithelium, even when derived from clinically inactive regions, possesses an inherent susceptibility to inflammatory stimuli, leading to impaired homeostatic functions (Karakasheva et al., 2026).

Upon inflammatory stimulation, CD enteroids showed further increases in cell death and an additional ~50% reduction in TEER. However, aside from these baseline differences, CD enteroids and enteroids from health subjects responded similarly to inflammation, exhibiting decreased proliferation, occludin, and F-actin expression. Previous studies suggest that enteroids from IBD patients (active or inactive areas) retain persistent transcriptional changes affecting barrier function, antimicrobial defense, and absorptive/secretory capacity due to epigenetic alterations in intestinal stem cells, likely explaining the abnormalties observed in our inactive CD enteroids at baseline and after inflammation (Planell et al., 2013; Dotti et al., 2017; Yoo and Donowitz, 2019; Woznicki et al., 2021). Specifically, recent evidence highlights the existence of inflammatory secretory progenitor (ISP) cells in the CD epithelium, which are hypersensitive to stimuli due to increased chromatin accessibility at ISP gene loci. This epigenetic memory is pre-established in CD intestinal stem cells, ensuring that even ex vivo expanded enteroids from inactive areas, such as the CDiE used in our study, maintain a “poised” state for excessive pro-inflammatory responses (Karakasheva et al., 2026). Furthermore, recent findings indicate that the IGFBP3/TMEM219 pathway is abnormally activated at the transcriptional level in CD intestinal stem cells, which triggers stem cell apoptosis and subsequently impairs mucosal healing (Amabile et al., 2025; D'Addio et al., 2025).

In contrast to enteroids, myofibroblasts from inactive CD ileum closely resembled healthy myofibroblasts, showing similar baseline and conditioned media-induced proinflammatory responses. Additionally, both healthy and inactive CD myofibroblasts exhibited comparable suppression of profibrotic genes in response to conditioned media, with the exception that CD myofibroblasts showed lower baseline expression of specific profibrotic markers (*COL1A1* and *ACTA2*), but not *TGFB* or *SRF* (**Figure 5B**; **Supplementary Figure 5C**).

The combined enteroid–myofibroblast model represents the early post-ileal resection state in fibrostenotic CD, suggesting that fibrogenesis is not yet activated at this stage. Further studies could evaluate if prolonged or additional cytokine exposures induce fibrogenesis, potentially identifying factors that prevent fibrosis progression. While this study focuses on inactive CD ileum post-resection, the model can be extended to investigate effects of ongoing inflammation, including whether conditioned media from active CD enteroids differentially affect healthy versus inactive CD myofibroblasts.

Our current model, using separate culture of ileal enteroids and subepithelial myofibroblasts (SEMF) with conditioned media transfer, allows controlled assessment of epithelial-to-mesenchymal signaling but lacks bidirectional epithelial–stromal interactions. Recent organ-on-a-chip studies co-culturing IBD epithelium and fibroblasts under physiological flow conditions demonstrated that stromal fibroblasts, rather than epithelial cells, drive barrier dysfunction, inflammation, and immune cell trafficking, highlighting the need for integrated models to fully capture epithelial–mesenchymal crosstalk (Özkan et al., 2024). Similarly, intestinal fibrosis in CD may be driven primarily by non-epithelial components, e.g., macrophages or neutrophiles. In our study using enteroids to model fibrosis initiation post-ileal resection, we identified changes in healthy and CD enteroids (both baseline and after damage) as unlikely primary initiators of fibrosis. These findings highlight the critical need to consider reciprocal epithelial–mesenchymal interactions, ideally through integrated co-culture platforms. Future studies using organ-on-a-chip systems, enabling dynamic epithelial–stromal communication, may better clarify the role of epithelial damage in CD-associated fibrosis and inflammation.

In IBD patients, Th1- type cytokines TNF and IFN-γ are elevated in the mucosa, driving inflammatory processes that synergistically increase IEC cell death and compromise epithelial barrier integrity (Bruewer et al., 2003; Woznicki et al., 2021). This study demonstrates that TNF plus IFN-γ exposure induces epithelial damage (increased cell debris and death) in all ileal enteroids (HE, CDiE, and CDaE). Damage was more pronounced in CD-derived enteroids (CDiE and CDaE), which exhibited higher cell debris intensity and PI-positive cell counts, indicating increased susceptibility to cytokine-induced cytotoxicity (**Figure 1**; **Supplementary Figure 2**). Consistent with this, previous studies reported significantly lower MTT viability and predominant necroptosis (rather than apoptosis) in TNF-exposed CD enteroids compared to healthy enteroids (Lee et al., 2021, 2022).

Surprisingly, inflammation markedly impacted cytoskeletal-related measurements, notably reducing TEER specifically in inactive CD enteroids but not in healthy enteroids (**Figure 4**). Additionally, inactive CD enteroids exhibited reduced F-actin expression both at baseline and after inflammatory stimulation (**Figure 3**), consistent with some prior studies but not all (Biskou et al., 2022; Jelinsky et al., 2023). However, occludin expression was not reduced in inactive CD enteroids compared to controls (**Figure 3**), in contrast to previous reports showing reduced occludin in inactive CD and ulcerative colitis colonoids (d'Aldebert et al., 2020). Given the significance of increased intestinal permeability in CD progression, we propose that the occludin-independent reduction in TEER, potentially linked to disrupted apical cytoskeleton (F-actin, myosin), warrants investigation as a therapeutic target.

In this study, conditioned media from cytokine-exposed HE and CDiE promoted inflammatory responses while suppressing fibrosis-associated gene expression in subepithelial myofibroblasts (**Figure 5**; **Supplementary Figure 4**). These findings suggest that, in early CD inflammation, damaged intestinal crypts may not directly initiate fibrogenesis. Damaged epithelial cells are hypothesized to stimulate fibrogenesis by activating nearby myofibroblasts (Shephard et al., 2004; Aden et al., 2010; Prunotto et al., 2012; Drygiannakis et al., 2013; Sakai and Tager, 2013; Tan et al., 2016); with the effect mediated through damage-associated molecular patterns (DAMPs) like TNF, IL-1α, and IL-33 (Valatas et al., 2017). Supporting this hypothesis, a previous study showed that conditioned medium from colonic epithelial cell lines (CaCo-2 and HT29) exposed to proinflammatory cytokines (TNF, IFN-γ, and IL-1α) significantly increased collagen production in HIMFs derived from both healthy and CD-active colonic tissue (Drygiannakis et al., 2013).

Unlike these previous studies, our findings surprisingly showed that conditioned media from ileal enteroids exposed to TNF plus Interferon-γ suppressed profibrotic gene expression in HIMF, regardless of whether the enteroids or HIMF were derived from healthy or inactive CD tissues (**Figure 5B**). While this finding of epithelial inflammatory response reducing fibrosis was in the setting of modeling the post-ileal resection state in CD, what occurs in intact tissue is likely more complex. Our findings suggest a novel ‘epithelial-to-mesenchymal’ inhibitory axis, where the secretome from acutely damaged enteroids acts as a protective feedback signal. This mechanism may temporarily prioritize epithelial restitution and reparative processes over excessive collagen deposition during the early phase of injury. Supporting this hypothesis, we observed an inductive trend of *SMAD7*, a key intracellular inhibitor of TGF-β signaling, in myofibroblasts exposed to the enteroid secretome (**Figure 5B**). The induction of *SMAD7* directly correlates with the simultaneous suppression of major profibrotic genes such as *COL1A1* and *ACTA2*, suggesting a regulated anti-fibrotic response originating from the epithelium to modulate fibroblast activation. The effects of other factors should be considered as potentially occurring in intact tissue and might explain the discrepancy between our findings and prior studies reporting epithelial-mediated activation of myofibroblasts.

Most importantly, we used a reductionist model, while intact intestine contains multiple other cell types and the microbiome, both of which are known as major contributors to CD pathogenesis. Macrophages and T helper cells potentially secrete pro-fibrogenic factors such as TGF-β1, IL-17, and IL-21 that further modulate the fibrotic response (Yoo et al., 2020). Tumor necrosis factor-like cytokine 1A and its receptor (TL1A–DR3) signaling activate fibrogenic pathways in CD4^+^ T cells, HIMFs, and IECs (Shih et al., 2009; Zheng et al., 2013; Shih et al., 2014; Bamias et al., 2017). Microbial ligands like flagellin from adherent-invasive E. coli (AIEC) activate IL-33/ST2 signaling in epithelial cells, promoting intestinal fibrosis (Imai et al., 2019; Watanabe and Kamada, 2022). Other differences in our studies and previous report include: 1) Our model used indirect interaction through conditioned media, without direct epithelial–myofibroblast contact. In other systems such as skin fibrosis, physical proximity between epithelial cells and fibroblasts was critical for myofibroblast activation (Jakubzick et al., 2004). 2) The cytokine exposure in our study was short-term (6 hours with TNF and IFN-γ). In prior studies only prolonged or sequential cytokine exposure—such as TNF followed by TGF-β1—effectively triggered profibrotic reprogramming in colonoids (Laudadio et al., 2024).

This model of post-ileal resection in CD, in which myofibroblasts derived from inactive CD ileum behaved similarly to those from healthy intestine—except for a partial reduction in basal profibrotic gene expression—resembles a previous report showing that myofibroblasts from inactive, non-strictured CD exhibited characteristics similar to those from healthy intestine (Di Sabatino et al., 2009). In contrast, myofibroblasts from active CD or inactive but strictured CD mucosa show enhanced fibrogenic activity. Intestinal fibroblasts from active CD secreted more collagen than healthy fibroblasts (Lawrance et al., 2001), and HIMFs from inactive, strictured CD expressed higher TGF-β, increased phosphorylated Smad2/3, reduced Smad7, and produced more collagen than controls (Di Sabatino et al., 2009). These differences highlight the need to characterize HIMFs across different CD states (inactive, active, strictured, non-strictured) to identify factors that shift the early post-resection, low-fibrosis state toward one that drives fibrogenesis.

Despite the novel insights provided by our human-derived model of epithelial-mesenchymal crosstalk, this study has several limitations. First, the number of CD-derived donor lines was limited (n=1 each for active and inactive CD), which limits the generalizability of our findings. However, each patient-derived enteroid was treated as a stable, unique cell line, similar to established models like Caco-2 or HT29, to ensure consistent disease phenotypes, supported by high technical reproducibility (3–6 independent experiments) and published evidence of conserved epigenetic memory in CD stem cells (Karakasheva et al., 2026). To ensure maximum transparency, all individual data points are shown in figures, and full line and passage details are provided in **Supplementary Table 1**. Second, while the conditioned media (CM) significantly modulated HIMF gene expression, the specific effector factors remain to be directly identified. Literature suggests that damaged epithelial cells release alarmins like IL-33 or IL-1α, the latter being a primary driver of proinflammatory activation in intestinal fibroblasts, which likely contribute to the observed proinflammatory cytokine induction in HIMF (Manetti et al., 2010; Nishida et al., 2010; Guo et al., 2014; Scarpa et al., 2015; Aggeletopoulou et al., 2022). Third, although this study provides comprehensive transcriptional profiles, further protein-level validation (e.g., ELISA, Western blot, or immunofluorescence) was not performed due to the limited availability of these specific patient-derived primary materials. Fourth, although our current model focused on identifying the initial signaling crosstalk via CM, we have not yet evaluated the effects of pharmacological interventions, such as anti-TNF or other immunosuppressants, within this system. Testing whether these agents can modulate or rescue the epithelial-mesenchymal interplay will be a critical next step to validate this platform as a tool for personalized medicine and drug screening. As a foundational pilot study, our work prioritizes establishing a robust ex vivo platform for studying intestinal epithelial-mesenchymal crosstalk. Future larger-scale studies with expanded donor cohorts, kinetic protein expression analysis, and proteomic characterization of the CM will be essential to further validate these mechanisms.

## 5 Conclusion

While epithelial damage in other organ fibrosis models is known to activate adjacent myofibroblasts via profibrotic mediators, our study found that conditioned media from TNF and IFN-γ-exposed healthy and inactive CD enteroids instead suppressed profibrotic gene expression in subepithelial myofibroblasts, modeling the early post-ileal resection state in CD.

This is the first study using primary human ileal epithelium to suggest that factors released from damaged crypts may independently modulate myofibroblast activity. Our findings indicate a potential early response where epithelial-derived signals do not necessarily promote, but may instead attenuate, specific profibrotic markers under certain conditions. Further validation with expanded cytoskeletal markers, such as α-SMA, and larger donor cohorts will be essential to confirm these observations. This model offers a platform to explore what additional factors convert this early antifibrotic state into the progressive fibrotic state that so often leads to addional strictures in CD and then to identify therapeutic targets to prevent fibrosis progression and reoperation in CD.

## Data Availability

The datasets presented in this study can be found in online repositories. The names of the repository/repositories and accession number(s) can be found in the article/**Supplementary Material**.
