# Supplementary material for: A model for Crohn’s disease post-ileal resection fibrosis development using human ileal enteroids and myofibroblasts
Source: Front Physiol. 2026 Apr 15;17:1764088. doi: 10.3389/fphys.2026.1764088 (PMC13126562; doi:10.3389/fphys.2026.1764088)
Supplement: Supplementary file 1 [file SupplementaryFile1.docx]

Supplementary Material

# Supplementary Data

**Supplementary Data Sheet 1 – Generative AI Use Log**

Tool: ChatGPT-4 (OpenAI)

Version: April 2025

Purpose: English language editing and grammar refinement

**Sample Prompts and Outputs**

**Prompt 1:**

Please revise the following discussion to make it more concise, while retaining all references.

Approximately half of CD patients with ileal disease undergo intestinal resection within 10 years of diagnosis (Frolkis et al., 2013; Frolkis et al., 2014). Along with fistulae and abscesses, stricturing complications account for to 40–70% of surgeries within the first 10 years following CD diagnosis (Rieder et al., 2013; Sleiman et al., 2021). Postoperative recurrence of strictures is common, particularly at the ileocolonic anastomotic site (Rutgeerts et al., 1990). The 10-year risk of requiring re-operative management after initial resection for CD is estimated to be around 35% (Frolkis et al., 2014). Controlling the ileal inflammation has not been shown to prevent stricture recurrence, although the timing of control of the inflammation after surgical resection and correlation with ileal fibrosis has not been definitely demonstrated.

**AI Output:**

Approximately half of CD patients with ileal disease require intestinal resection within 10 years of diagnosis (Frolkis et al., 2013; Frolkis et al., 2014). Stricturing complications account for 40–70% of these surgeries (Rieder et al., 2013; Sleiman et al., 2021), and recurrence, especially at ileocolonic anastomoses, is common (Rutgeerts et al., 1990). About 35% of patients require repeat surgery within 10 years (Frolkis et al., 2014). However, whether controlling ileal inflammation post-surgery effectively prevents stricture recurrence remains unclear.

**Prompt 2:**

I would like to present the following as the conclusion. Please revise it for grammar and conciseness while retaining all key points.

In various established organ fibrosis models, it is known that damaged epithelial cells release profibrotic mediators that activate nearby myofibroblasts, thereby contributing to organ fibrosis. In this study, we investigated the profibrotic effects of conditioned media from TNF and IFN-γ (T+I)-exposed healthy and inactive CD ileal enteroids (CDiE) on subepithelial myofibroblasts (SEMF), modeling early post-ileal resection conditions in Crohn’s disease. This is the first study to use primary human intestinal epithelium, and in contrast to previous findings, we observed that damaged ileal crypts actually suppressed profibrotic gene expression in pericryptal subepithelial myofibroblasts. Morphologically, there was also a subtle trend of myofibroblasts shifting from a spindle-like to a stellate-like shape. These findings suggest that damaged intestinal epithelial cells (IECs) may release mediators that de-activate rather than activate fibroblasts. This may support previous observations in CD mucosa showing a loss of pericryptal α-SMA-positive myofibroblasts (illustrated in **Fig. 7**). However, due to the lack of immunofluorescence data (e.g., α-SMA), further validation of this phenotype is required.

This model of the early post-ileal resection state provides a platform to identify key targets for preventing fibrosis progression and potentially reducing the need for repeat ileal surgery in CD patients.

**AI Output:**

while epithelial damage in other organ fibrosis models is known to promote myofibroblast activation via profibrotic mediators, our study—using primary human ileal enteroids—found that conditioned media from TNF and IFN-γ-exposed healthy and inactive CD enteroids (CDiE) suppressed profibrotic gene expression in subepithelial myofibroblasts (SEMF), modeling the early post-ileal resection state in Crohn’s disease. This is the first study using primary ileal epithelium to show that damaged crypts may reduce rather than enhance myofibroblast fibrogenic activation. Morphologically, HIMFs showed a subtle shift from a spindle-like to a stellate-like appearance, suggesting a possible phenotypic inactivation, rather than activation, driven by IEC-derived mediators. These findings align with reports of loss of pericryptal α-SMA⁺ myofibroblasts in CD mucosa and are illustrated in **Fig. 7.** However, due to the lack of immunofluorescence data (e.g., α-SMA), further validation is required.

This model provides a platform to investigate early antifibrotic signals and identify therapeutic targets to prevent fibrosis progression and reduce the need for repeat ileal surgery in CD.

**Please note that Figure 7 was removed as we felt that the data was too preliminary to include.**

# Supplementary Figures and Tables

## Supplementary Figures

**Supplementary Fig. 1**

**Representative Images of 3D Enteroids and Characterization of HIMFs.**

(A): 3D Enteroids: Representative phase-contrast images of 3D enteroids derived from healthy controls and patients with inactive CD. These fully developed 3D structures were used to generate epithelial monolayers. *Scale bar: 100 µm.*

(B): HIMFs (Human Intestinal Myofibroblasts): Representative images of HIMFs isolated from healthy controls and inactive CD ileum at baseline. The cells exhibit the characteristic spindle-shaped morphology of myofibroblasts. *Scale bar: 100 µm.*

(C): Immunophenotype characterization of HIMF: To confirm the identity of isolated HIMFs, cells were seeded onto chamber slides and stained for specific markers. HIMFs were identified as α-SMA-positive (green) and vimentin-positive (red), while being desmin-negative. Nuclei were counterstained with DAPI (blue). Images were acquired using a confocal laser scanning microscope. Negative control: DAPI staining only. *Scale bar: 200 µm.*

**Supplementary Fig. 2**

**Effect of TNF and IFN-γ on cell death in ileal enteroid monolayers derived from active Crohn’s disease.**

(A): Phase contrast images of ileal enteroid monolayers after 6-hour exposure of T+I. The images show both unexposed and exposed monolayers. Conditions represented are: HE_Ctrl (healthy enteroid control, no exposure), HE_T+I (healthy enteroid with TNF and IFN-γ exposure), CDaE_Ctrl (CD active enteroid control, no exposure), and CDaE_T+I (CD active enteroid with TNF and IFN-γ exposure). *Scale bar: 200 µm*.

(B, C): Following 6-hour T+I exposure plus an additional 18-hour conditioning, phase contrast (B) and propidium iodide fluorescence images (C) of ileal enteroid monolayers were captured for the above four conditions (*Scale bar: 200 µm*). Images in supplementary Fig. 2A-2C are representative of similar results in 2 independent experiments. Enlarged views of the representative regions (indicated by white boxes) are displayed below each corresponding propidium iodide image.

(D): Cell debris intensity post 6-hour exposure was determined using ImageJ software in 12 different same sized surface areas (16,480 µm^2^) from 2 independent experiments.

(E): Cell debris intensity following 6-hour exposure plus 18-hour conditioning was determined using ImageJ software in 12 different same sized surface areas (16,480 µm^2^) from 2 independent experiments.

(F): Propidium Iodide (PI)-positive cell counts were determined using ImageJ software in 12 different same sized surface areas (16,480 µm^2^) from 2 independent experiments.

(D-F): Results presented as means ± standard error of the mean (SEM), with statistical analysis performed using ANOVA followed by Tukey’s *post-hoc* testing. Enteroid lines used for phase contrast images and PI staining include HE (n=1; 109I) and CDaE (n=1; 68I).

**Supplementary Fig. 3**

**Effect of TNF and IFN-γ on epithelial permeability in ileal enteroid monolayers derived from active Crohn’s disease.**

(A): TEER of ileal enteroid monolayers were measured at 3 different time points – baseline, after 6-hour exposure of T+I, and after 6-hour exposure of T+I plus 18-hour conditioning for all groups (HE_Ctrl, HE_T+I, CDaE_Ctrl, and CDaE_T+I).

(B): The percentage changes in TEER between two time points (baseline and after 6-hour exposure) were calculated and plotted for all groups.

(C): The percentage changes in TEER between two time points (baseline and after 6-hour exposure of T+I plus 18-hour conditioning) were calculated and plotted for all groups.

(A-C): Data are from 3 independent experiments. For TEER measurement, the enteroid lines used were HE (n=2; 109I, 104I) and CDaE (n=1; 68I). Error bars represent the SEM, and statistical significance was determined using ANOVA followed by Tukey’s *post-hoc* test.

**Supplementary Fig. 4**

**Effects of cytokine exposed enteroid-derived conditioned medium on pro-inflammatory gene transcription in human ileal myofibroblasts (3-hour incubation)**

**The short-term effect (3 hours) of cytokine exposed enteroid-derived conditioned media on HIMF was investigated using the same experimental design illustrated in Fig. 1A.**

After a 6-hour exposure with T+I, followed by an 18-hour conditioning, the resultant basolateral conditioned medium was then exposed to HIMF for 3 hours. We analyzed differential gene expression profiles in HIMF-HC and HIMF-CD for all groups. Quantitative PCR was utilized to assess relative mRNA levels of proinflammatory genes (*IL1B*, *MCP1*, and *IKBA*). Data were normalized to *GAPDH*. The results are presented as means ± SEM, and statistical significance was determined using ANOVA followed by a Tukey’s *post-hoc* test for 4 independent experiments. For quantitative PCR, the enteroid lines used were HE (n=1; 60I) and CDiE (n=1; 69I).

**Supplementary Fig. 5**

**Effects of cytokine-unexposed enteroid-derived conditioned medium on proinflammatory and profibrotic gene transcription in human ileal myofibroblasts**

(A-C): mRNA expression pattern of proinflammatory and profibrotic genes in HIMF from HIMF-HC and HIMF-CD, following treatment with three media types - basal medium (BM), HE-conditioned medium, CDiE-conditioned medium. For HE-conditioned medium and CDiE-conditioned medium, the previous enteroid-derived conditioned media after 18-hour conditioning were used as illustrated in Fig. 1A. The three media were treated for 24 hours (A and C) and 3 hours (B). Quantitative PCR was utilized to assess relative mRNA levels of proinflammatory genes (*IL1B, IL6, MCP1,* and *IKBA*), profibrotic genes (*COL1A1, ACTA2,* and *TGFB*), and anti-fibrotic gene *SMAD7*. Data were normalized to *GAPDH*. Presented data show means ± SEM, derived from 3 independent experiments. Statistical significance was determined using ANOVA, followed by a Tukey’*s post-hoc* test. For quantitative PCR, the enteroid lines used were HE (n=1; 60I) and CDiE (n=1; 69I).

## Supplementary Tables

**Supplementary Table 1. Enteroid Line and Passage Summary**

| **Figure** | **Independent exam** | **Enteroid (line and passage)** |
| --- | --- | --- |
| Fig. 1B and 1E | 4 | 60I P34 vs 69I P16 60I P36 vs 69I P18 60I P40 vs 69I P22 46I P22 vs 69I P8 |
| Fig. 1C and 1F | 5 | 60I P34 vs 69I P16 60I P36 vs 69I P18 60I P36 vs 69I P18 46I P22 vs 69I P8 109I P41 vs 69I P14 |
| Fig. 1D and 1G | 3 | 60I P36 vs 69I P18 46I P22 vs 69I P8 109I P41 vs 69I P14 |
| Fig. 2 (Ki67) | 3 | 60I P40 vs 69I P22 60I P40 vs 69I P22 46I P22 vs 69I P8 |
| Fig. 3B (Occludin) | 3 | 60I P36 vs 69I P18 60I P36 vs 69I P18 60I P40 vs 69I P22 |
| Fig. 3C (F-actin) | 3 | 60I P36 vs 69I P18 60I P36 vs 69I P18 46I P22 vs 69I P8 |
| Fig. 3D (Cell height) | 5 | 60I P36 vs 69I P18 60I P36 vs 69I P18 60I P40 vs 69I P22 60I P40 vs 69I P22 46I P22 vs 69I P8 |
| Fig. 4 (TEER) | 5 | 60I P36 vs 69I P18 60I P36 vs 69I P18 60I P40 vs 69I P22 46I P22 vs 69I P8 109I P41 vs 69I P14 |
| Fig. 5 (PCR, 24h) | 6 | 60I P30 vs 69I P10 60I P30 vs 69I P10 60I P32 vs 69I P14 60I P34 vs 69I P16 60I P36 vs 69I P18 46I P22 vs 69I P8 |
| Supple Fig. 2 | 2 | 109I P41 vs 68I P34 109I P41 vs 68I P34 |
| Supple Fig. 3 (TEER) | 3 | 109I P41 vs 68I P34 109I P41 vs 68I P34 104I P38 vs 68I P34 |
| Supple Fig. 4 (PCR, 3h) | 4 | 60I P34 vs 69I P16 60I P34 vs 69I P16 60I P36 vs 69I P18 60I P40 vs 69I P22 |
| Supple Fig. 5A (PCR, 24h) | 3 | 60I P34 vs 69I P16 60I P36 vs 69I P18 60I P40 vs 69I P22 |
| Supple Fig. 5B (PCR, 3h) | 3 | 60I P36 vs 69I P18 60I P36 vs 69I P18 60I P40 vs 69I P22 |
| Supple Fig. 5C (PCR, 24h) | 3 | 60I P34 vs 69I P16 60I P36 vs 69I P18 60I P40 vs 69I P22 |
