# Supplementary figures and images for: A model for Crohn’s disease post-ileal resection fibrosis development using human ileal enteroids and myofibroblasts
Source: Front Physiol. 2026 Apr 15;17:1764088. doi: 10.3389/fphys.2026.1764088 (PMC13126562; doi:10.3389/fphys.2026.1764088)

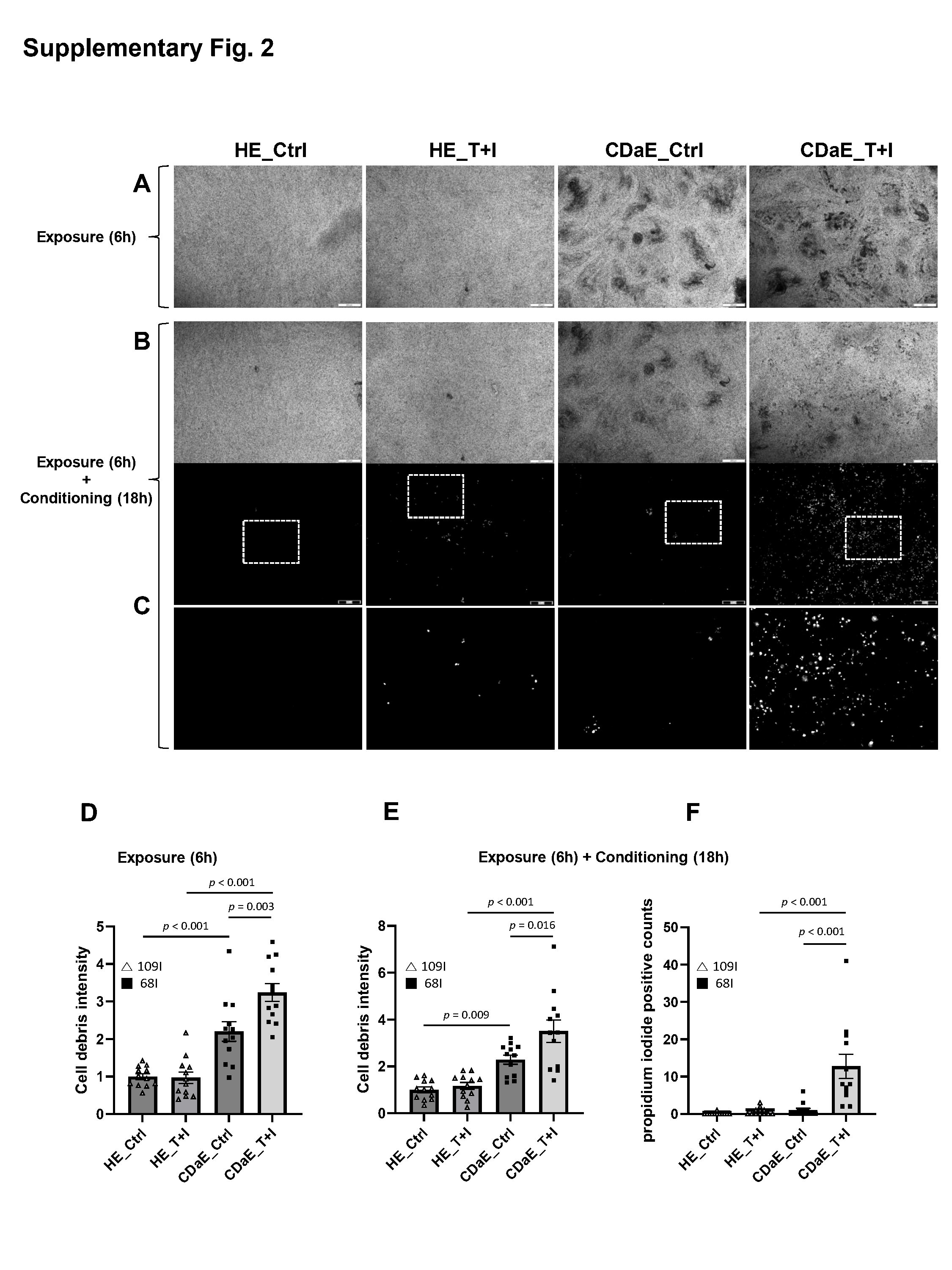

Supplement: Supplementary file 2 [file Image1.jpg]

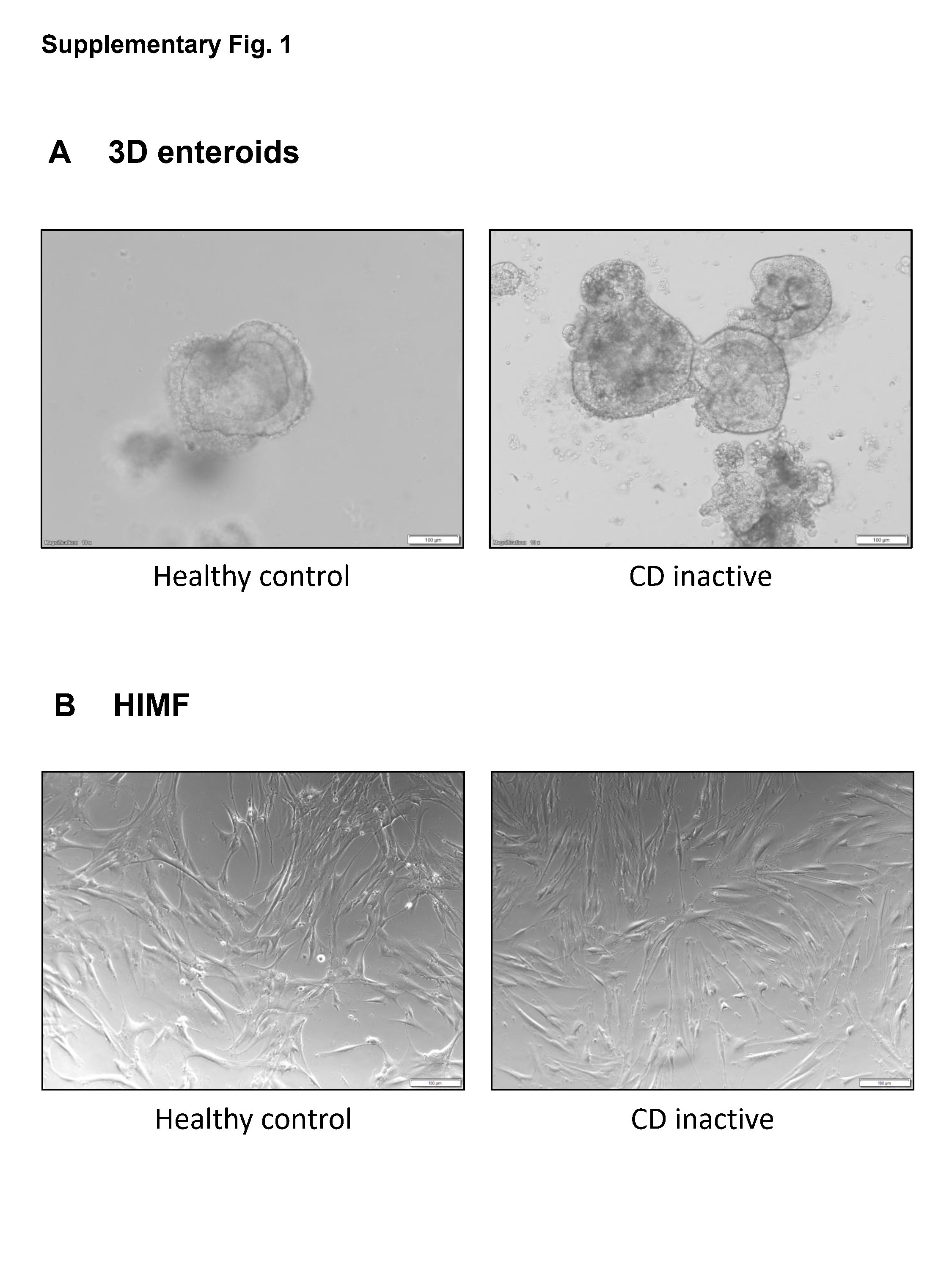

Supplement: Supplementary file 3 [file Image2.jpeg]

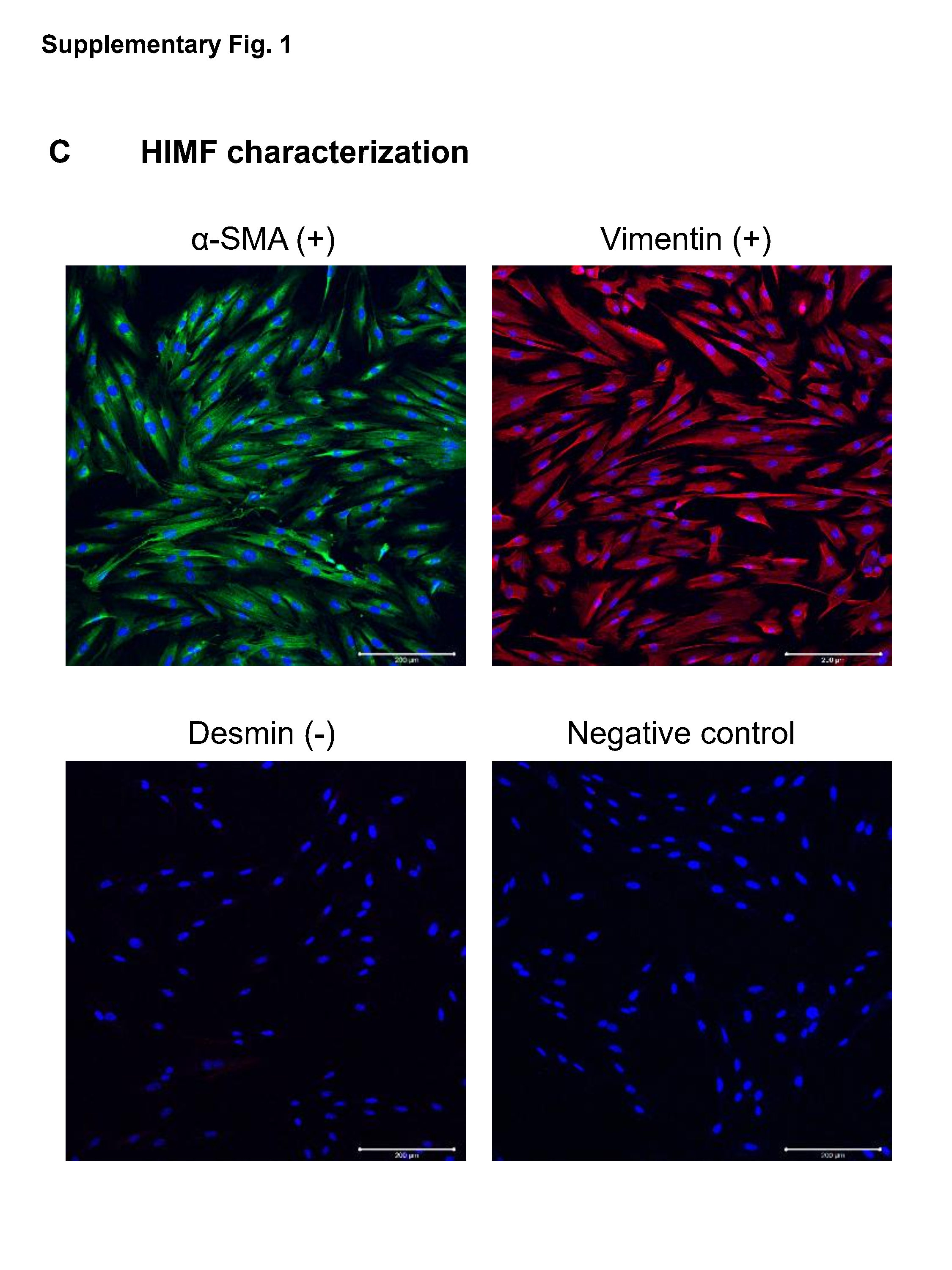

Supplement: Supplementary file 4 [file Image3.jpeg]

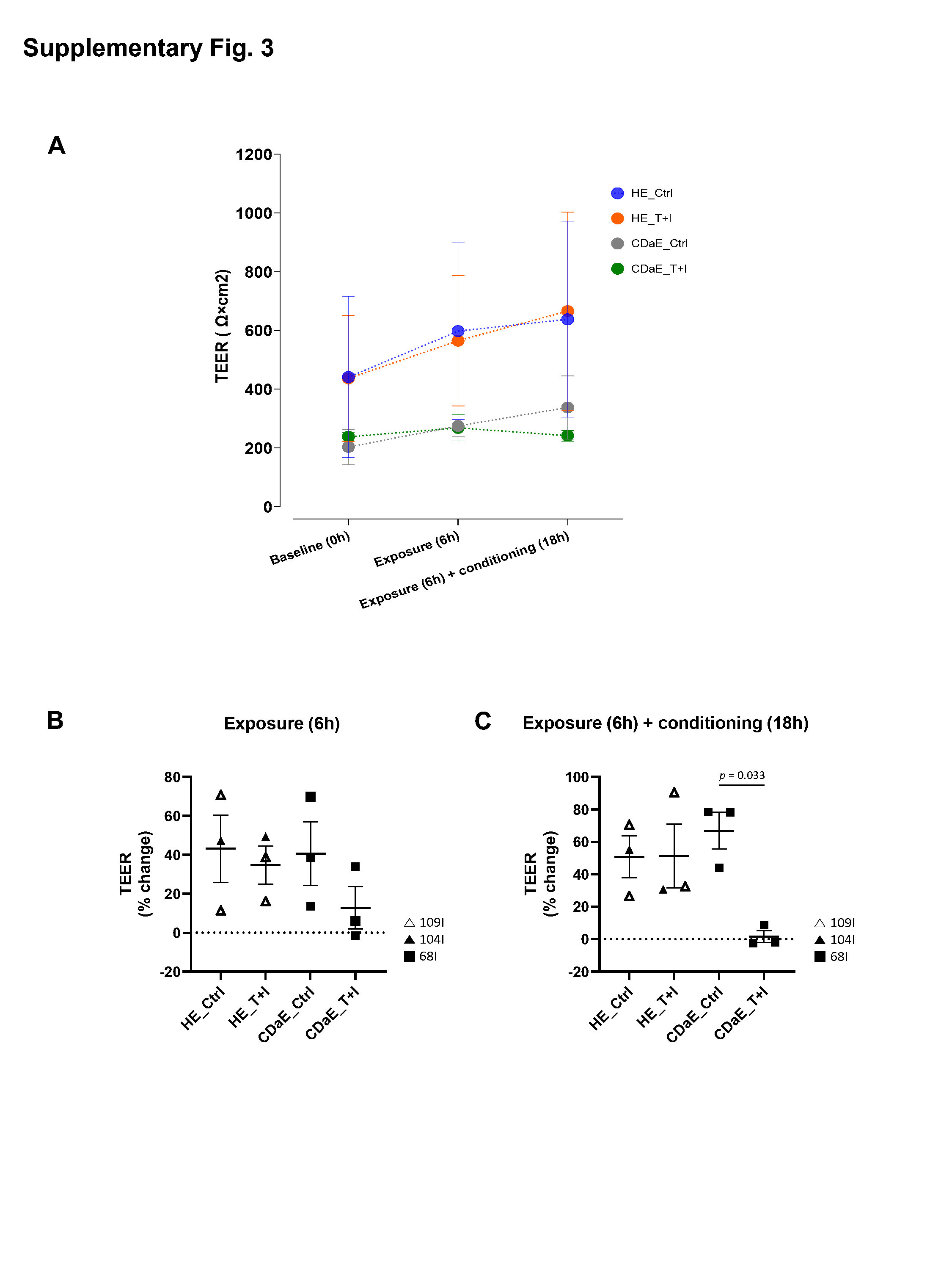

Supplement: Supplementary file 5 [file Image4.jpeg]

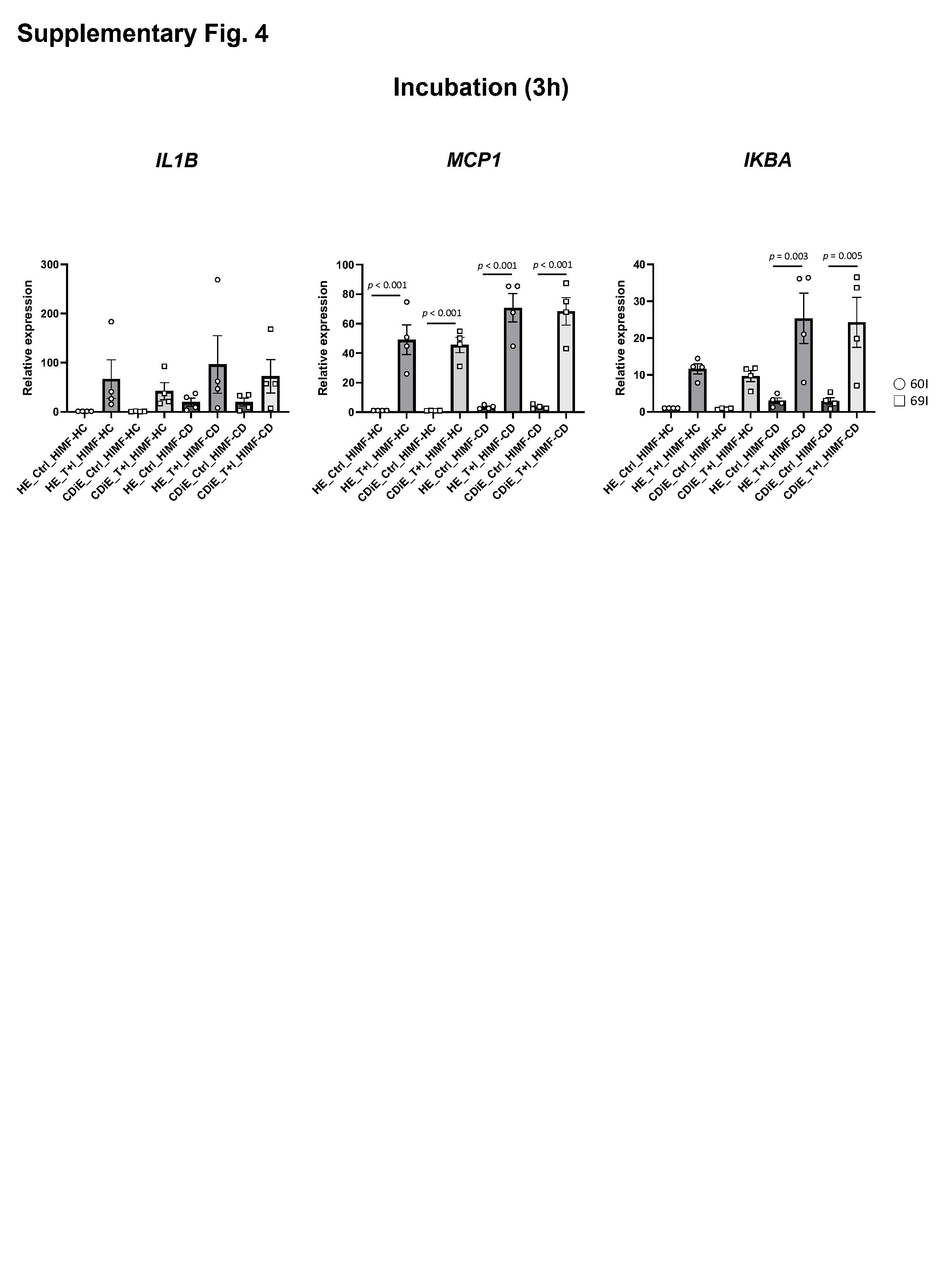

Supplement: Supplementary file 6 [file Image5.jpeg]

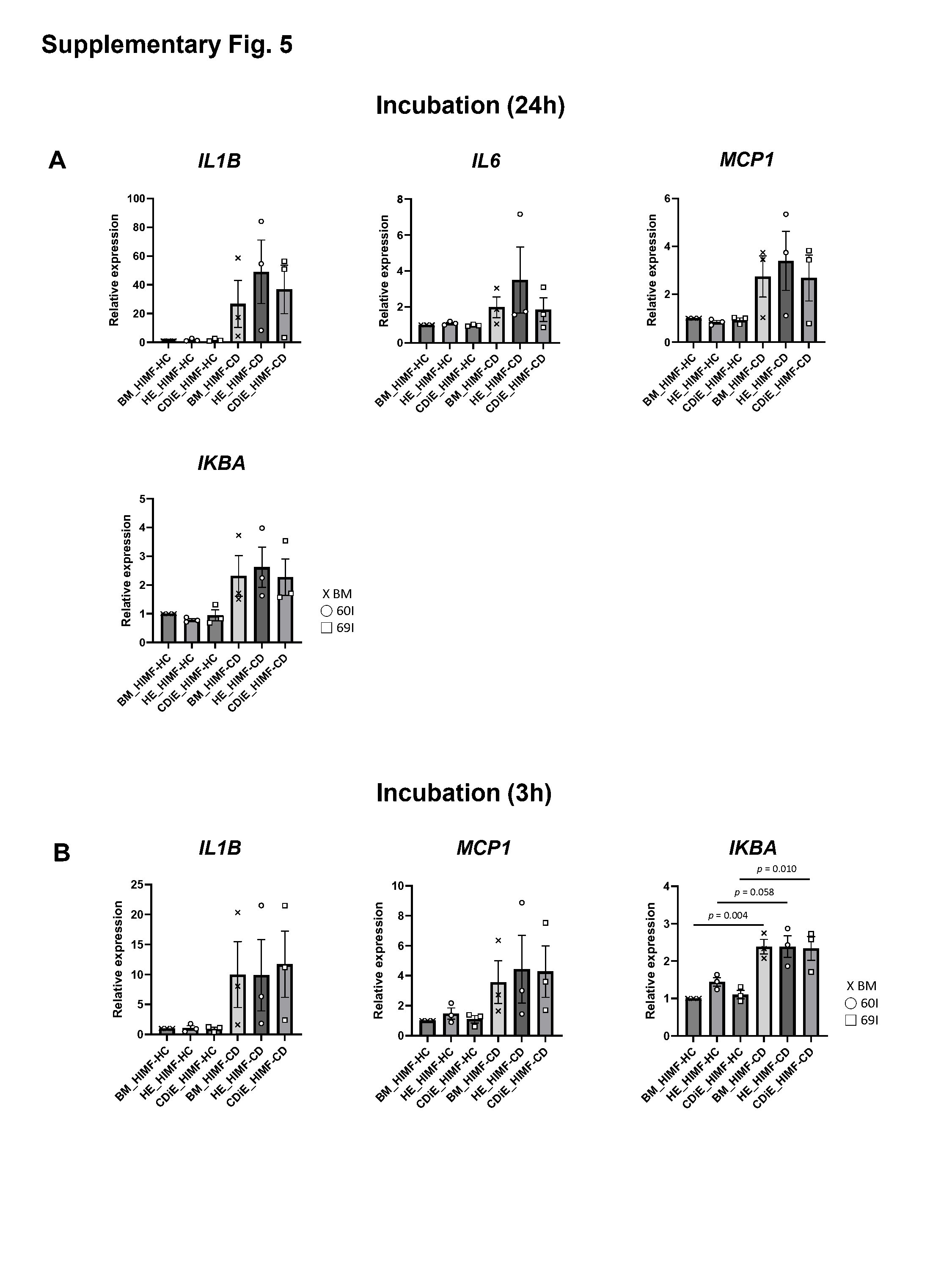

Supplement: Supplementary file 7 [file Image6.jpeg]

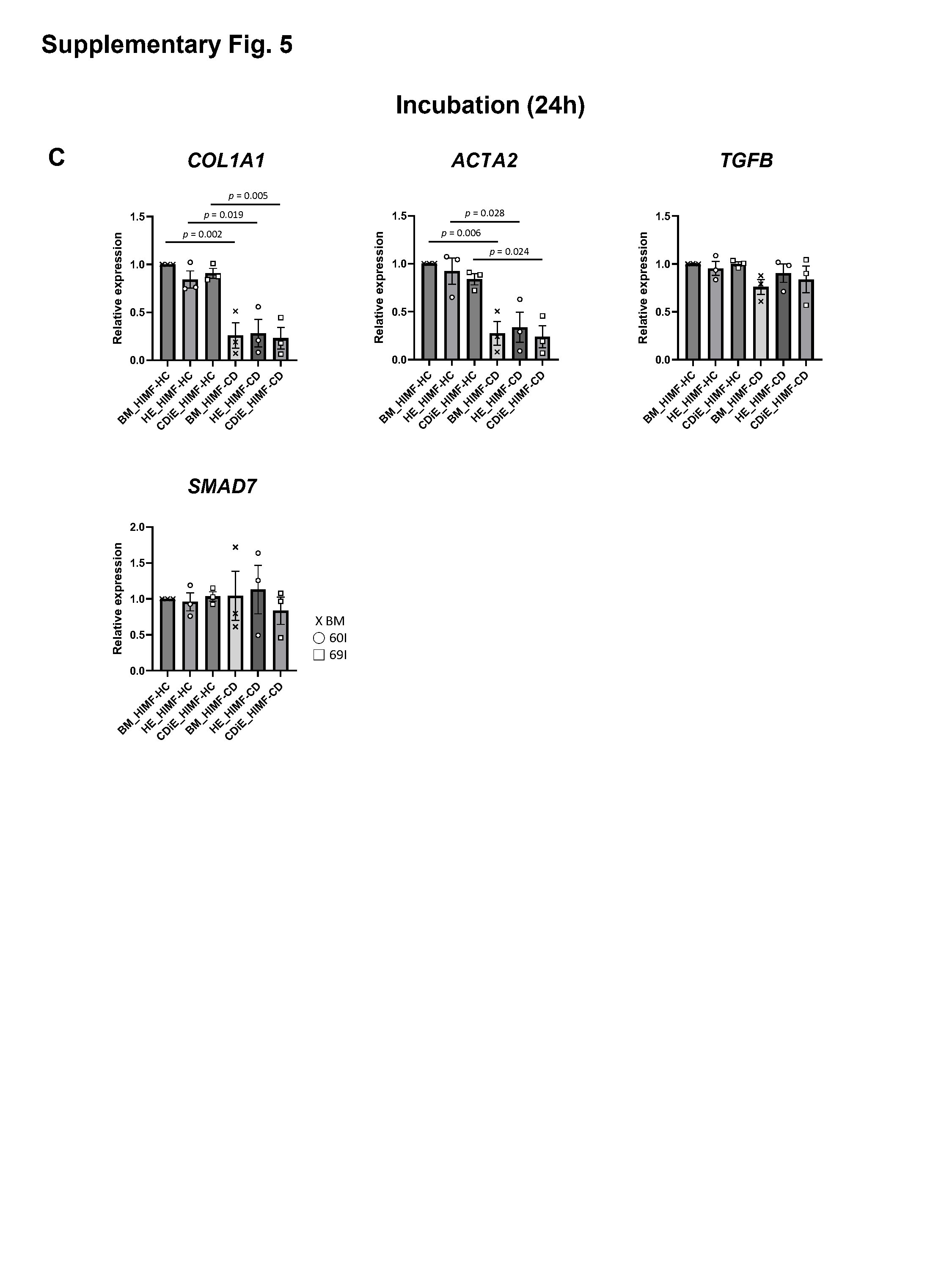

Supplement: Supplementary file 8 [file Image7.jpeg]
